# Shikonin as a therapeutic agent in renal cell carcinoma: insights from TEK-related causal association with glaucoma

**DOI:** 10.3389/fphar.2025.1580704

**Published:** 2025-07-30

**Authors:** Ruyue Jia, Yiran Liang, Benkui Zou, Xiangzhi Li, Tao Chen, Chao Zhang, Jiasheng Bian, Renbo Guo

**Affiliations:** ^1^ Department of Urology, Shandong Cancer Hospital and Institute, Shandong First Medical University and Shandong Academy of Medical Sciences, Jinan, China; ^2^ Shandong Provincial Key Laboratory of Precision Oncology, Cancer Research Center, Shandong Cancer Hospital and Institute, Jinan, Shandong, China; ^3^ Department of Breast Surgery, General Surgery, Qilu Hospital of Shandong University, Jinan, China; ^4^ Shandong Provincial Key Laboratory of Animal Cell and Developmental Biology, School of Life Sciences, Shandong University, Qingdao, China

**Keywords:** gene expression, glaucoma, Mendelian randomization (MR), NHANES, renal cell carcinoma (RCC), shikonin, TEK

## Abstract

**Introduction:**

Renal cell carcinoma (RCC) is a lethal malignancy with rising incidence, while glaucoma, a chronic eye disease, shares systemic mechanisms such as oxidative stress and inflammation with cancers. This study aimed to investigate the causal link between glaucoma and RCC and explore molecular intersections to identify novel therapeutic targets.

**Methods:**

A two-step Mendelian randomization (MR) analysis using genetic data from the NHGRI-EBI GWAS Catalog and FinnGen database was performed, supplemented by NHANES data. Gene expression analysis (GSE53757, E-MTAB-1980) identified glaucoma-related genes in RCC. Molecular docking and functional assays evaluated shikonin's effects on TEK and AKT/mTOR signaling.

**Results:**

MR revealed a significant causal relationship between glaucoma and RCC. TEK, a glaucoma-related gene, was downregulated in RCC tissues and correlated with advanced tumor stage and metastasis. Shikonin and acetylshikonin upregulated TEK expression, inhibited RCC cell proliferation/migration, and suppressed AKT/mTOR phosphorylation.

**Discussion:**

These findings support a role for glaucoma-associated genes in RCC development and progression, highlighting shikonin as a promising therapeutic agent targeting this molecular axis.

## 1 Introduction

RCC is a highly lethal malignancy, accounting for 80%–85% of primary renal tumors (Lu et al., 2024), with a mortality rate of 30%–40% (Bahadoram et al., 2022). In recent years, the incidence of RCC has continued to rise (Woon et al., 2024), posing a growing threat to public health and imposing a substantial economic burden. Despite advancements in surgical and targeted therapies, the prognosis for patients with advanced RCC remains poor, underscoring the urgent need for deeper biological insights and novel therapeutic strategies (Young et al., 2024).

Emerging evidence suggests that chronic diseases, including both systemic and organ-specific conditions, may share common pathophysiological mechanisms. Glaucoma, a leading cause of visual impairment, is increasing recognized not only as a localized ocular disorder but also as a condition associated with systemic pathophysiological mechanisms such as oxidative stress (Hsueh et al., 2022), mitochondrial dysfunction (Jassim et al., 2021), and inflammation (Baudouin et al., 2021), which are also implicated in the development and progression of cancer (van Noorden et al., 2024; Hallaj et al., 2021; Wildner, 2021). Notably, both RCC and glaucoma exhibit dysregulated cell death (Basavarajappa et al., 2023; He et al., 2024), inflammatory response (Kruk et al., 2023; Harun-Or-Rashid and Inman, 2018), and immune dysfunction (Hoppe and Gregory-Ksander, 2024; Braun and Chakraborty, 2023), raising the possibility of shared molecular pathways and a potential causal link between these two seemingly unrelated diseases.

Natural compounds, particularly those derived from traditional medicinal plants, have gained increasing attention for their multi-target anticancer properties and reduced toxicity profiles compared to synthetic agents (Chen et al., 2019). Among them, shikonin and its acetylated derivative acetylshikonin, extracted from Lithospermum erythrorhizon, have demonstrated potent anti-inflammatory, antioxidant, and anti-angiogenic effects (Sun et al., 2022). Recent studies have shown that these compounds inhibit tumor growth in various cancers by modulating key pathways such as STAT3, NF-κB, and PI3K/AKT (Tang et al., 2022; Gong and Li, 2011). However, their role in RCC, particularly in relation to glaucoma-associated molecular pathways, remains largely unexplored.

Given these observations, this study was designed to address three interconnected objectives: first, to investigate the potential causal relationship between glaucoma and RCC risk; second, to identify and functionally validate glaucoma-associated genes dysregulated in RCC, with a particular focus on TEK; and third, to evaluate the therapeutic potential of shikonin in targeting TEK and suppressing RCC progression via modulation of glaucoma-related signaling.

Mendelian randomization (MR) leverages genetic variants as instrumental variables (IVs), which typically precede the occurrence of diseases or phenotypes and are not influenced by subsequent environmental and behavioral factors (Pierce and Burgess, 2013), therefore less susceptible to confounding or reverse causation (Burgess et al., 2013), making it a robust method for inferring causality between exposure and outcome. Additionally, the National Health and Nutrition Examination Survey (NHANES) is a significant national health and nutrition examination survey, conducted continuously by the CDC in the United States (Gunter, 1997). It provides comprehensive data on the health and nutrition status of the American population, offering a unique resource for studying RCC in the context of glaucoma.

In this study, we employed a multi-stage, integrative approach combing epidemiological, genomic, and experimental methodologies. We conducted MR analysis using large-scale genetic datasets from the NHGRI-EBI GWAS Catalog and FinnGen database, supplemented by observational data from NHANES, revealing the causal relationship between glaucoma and RCC risk for the first time. Second, we performed multi-omics analysis to identify glaucoma-associated genes dysregulated in RCC tissues, with a particular focus on TEK. Finally, we investigated the therapeutic potential of shikonin, demonstrating its anti-tumor effects via modulation of the TEK-AKT/mTOR signaling pathways. By integrating population-level evidence with molecular mechanistic insights, our study not only reveals a novel glaucoma-RCC connection but also highlights shikonin as a promising agent for RCC treatment.

## 2 Materials and methods

### 2.1 GWAS data sources

Cataract and uveitis GWAS data were sourced from the National Human Genome Research Institute‐European Bioinformatics Institute (NHGRI‐EBI) GWAS Catalogue. Data for other exposure variables, including macular degeneration, diabetic retinopathy, conjunctivitis and keratitis, as well as the RCC outcome variable, were extracted from the FinnGen database. Glaucoma data were sourced from the NHGRI-EBI GWAS Catalog and FinnGen database for both stages of the MR analysis. The datasets used focused on European populations; further details are provided in Supplementary Table S1.

### 2.2 Selection of instrumental variables (IVs)

Single nucleotide polymorphisms (SNPs) associated with ocular diseases were selected from GWAS data. To ensure strong IVs, we adhered to the following criteria:(1) significant genome-wide associations (p < 5 × 10^−8^); (2) low linkage disequilibrium (r^2^ < 0.001, kb > 10,000); (3) strong instrument strength (F-value ≥10), minimizing the risk of weak instrument bias (Nikos et al., 2020). Detailed SNP information is presented in Supplementary Table S2.

### 2.3 MR analysis

**FIGURE 1:**
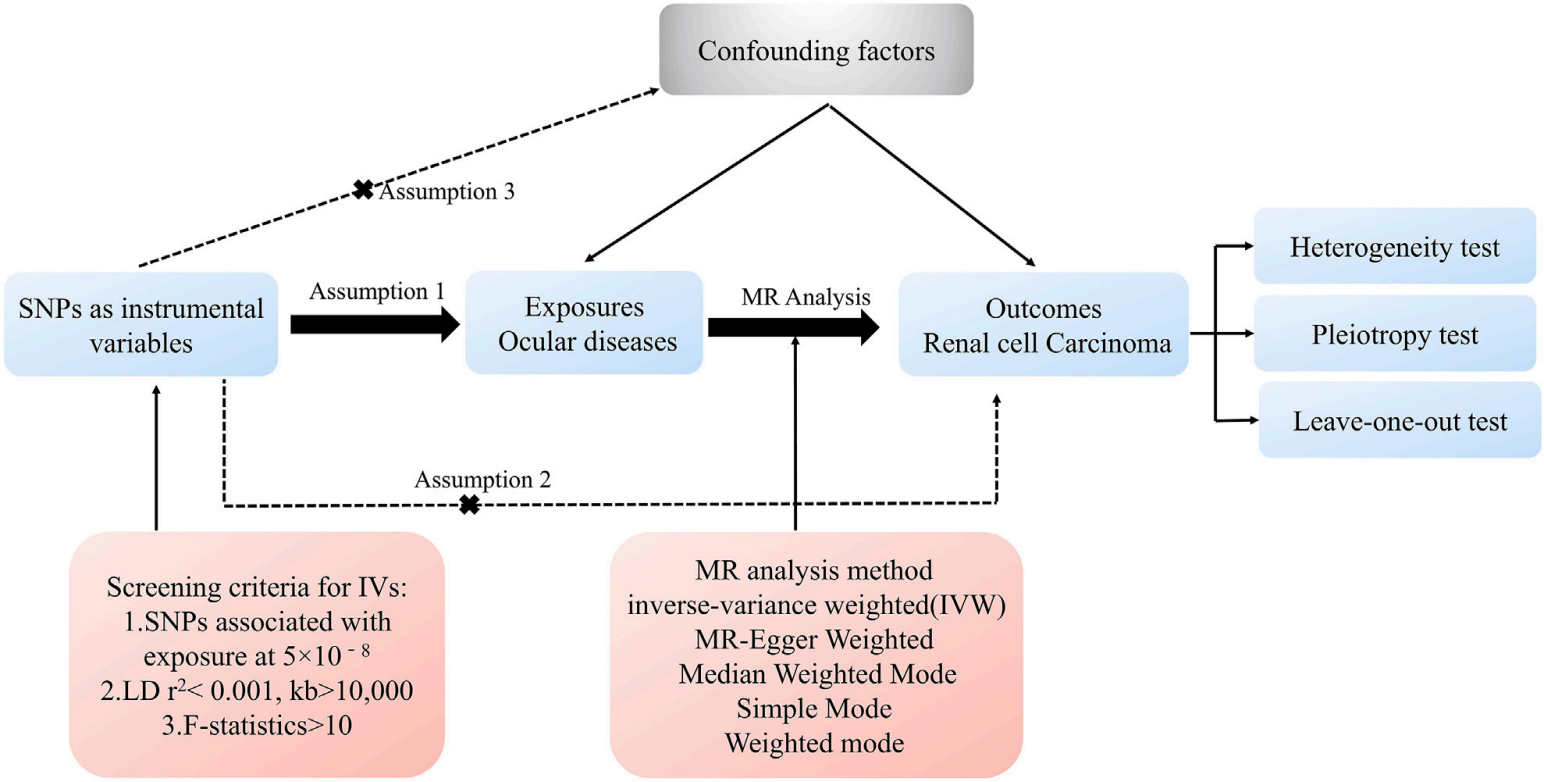
The progression of the MR study and its three principal assumptions.

We employed a two-step MR approach to evaluate the causal relationship between ocular diseases (e.g., glaucoma, cataract, macular degeneration, diabetic retinopathy, conjunctivitis,keratitis and uveitis), and RCC. In the first stage, we investigated the potential causal connection between various ocular disease and RCC risk. In the second stage, we focused specifically on glaucoma, incorporating data from additional databases. An overview of the analytical approach is shown in Figure 1. The IVW method, known for its high statistical power, was the primary analysis approach when all IVs are valid (Burgess and Thompson, 2013). Additional MR methods were employed to validate results and address potential biases.

Sensitivity analyses were conducted to assess the robustness of MR findings. Cochran’s Q test was used to assess heterogeneity, with p-values >0.05 indicating negligible heterogeneity impact (Yavorska and Burgess, 2017). MR-Egger intercept tests were conducted to detect horizontal pleiotropy, with p-values >0.05 suggesting a low risk of pleiotropic bias (Hartwig et al., 2017). Leave-one-out analyses were conducted to evaluate the influence of individual SNPs on the overall estimates (Hemani et al., 2018).

### 2.4 Study population in NHANES

The NHANES database filtered 7,003 participants from 2005 to 2008, all with comprehensive data. Urinary malignancies (UC) data were derived from the survey responses to the question, “What kind of cancer?”, which included “kidney cancer,” “bladder cancer,” or “prostate cancer.”

Our covariates included gender (female, male), age (>65, <65), race (Mexican American, Non-Hispanic Black, Non-Hispanic White, Other Hispanic, Other Race - Including Multi-Racial), education (9-11th grade, college graduate or above, high school graduate, less than 9th grade, some college or AA degree), hypertension (no, yes), overweight (no, yes), smoking (no, yes), and glaucoma (no, yes). Hypertension, overweight, glaucoma was defined by a positive response to the question: “Has a doctor or other health professional ever told you that you have hypertension/overweight/glaucoma?”. Covariate classifications were chosen to analyze significant correlations with UC occurrence, avoiding more complex classifications like quartiles.

### 2.5 NHANES analysis

A weighted logistic regression model was employed to analyze baseline characteristics and clarify relationships between covariates and UC. Subgroup analysis using the Chi-square test provided additional insights. Results were presented as β coefficients with 95% confidence intervals (95% CI). The interaction p-value between each covariate and glaucoma on UC was assessed, with values >0.05 indicating no significant differences in the causal relationship across covariate levels.

### 2.6 Identification of glaucoma-related genes

Based on prior literature (Kumar et al., 2024; Selvan et al., 2022; Christ et al., 2015), several key glaucoma-related genes were identified. Unpaired t-test was performed to compare gene expression levels in normal renal tissues and RCC tissues using the GSE53757 dataset.

### 2.7 Gene analysis and prognostic evaluation

Non-negative matrix factorization (NMF) clustering was used to analyze gene expression and patient survival rate data in the E-MTAB-1980 kidney dataset, grouping patients into high-risk and low-risk groups. Differential expression of glaucoma-related genes was compared across groups, and Cox regression analysis and Kaplan-Meier survival curves were used to assess their impact on RCC prognosis.

### 2.8 Compound screening and molecular docking

Using the HIT-index database (http://www.badd-cao.net:2345/), potential plant-derived compounds binding to TEK were predicted, with pyridine, shikonin, and its acetylated derivatives were selected for further analysis. The two-dimensional and three-dimensional chemical structures of these compounds were obtained from the PubChem database (https://pubchem.ncbi.nlm.nih.gov/). The TEK protein structure (UniProt ID: Q02763) was retrieved from the Protein Data Bank (PDB, https://www.rcsb.org/) and prepared using AutoDock Tools, involving removal of water molecules, addition of polar hydrogens, and conversion of both the receptor and ligands to PDBQT format. Molecular docking was performed with AutoDock Vina, configuring the receptor grid at Cartesian coordinates (1.812, 0.024, 21.293) Å with a 40 × 40 × 40 Å box. The energy search range was set at ±5 kcal/mol, generating 20 binding modes, with docking results sorted by binding energy.

### 2.9 RT-qPCR and western blotting

Total RNA was extracted from human normal kidney cell line HK2 and RCC cell lines 786-O and 769-P using TRIzol reagent (Ambion, 15596026). Reverse transcription was performed with Hifair^®^II 1st Strand cDNA Synthesis SuperMix for qPCR (YEASEN, 11123ES60) following the manufacturer’s protocol. Real-time quantitative PCR (RT-qPCR) was performed using gene-specific primers for TEK and β-actin (sequences detailed in Supplementary Table S3) with SYBR Green Master Mix (Servicebio, G3324-15) on a thermal cycler.

Western blotting was used to evaluate protein expression in transfected, drug-treated, and control cells, with GAPDH as the loading control. Equal amounts of total protein were mixed with loading buffer, separated by SDS-PAGE, and transferred to nitrocellulose membranes. Membranes were blocked with 5% non-fat dry milk, then incubated overnight at 4°C with primary antibodies against TEK (Proteintech, 19157-1-AP), AKT (Proteintech, 10176-2-AP), p-AKT (Proteintech, 66444-1-Ig), p-mTOR (Proteintech, 67778-1-Ig), and GAPDH (Proteintech, 60004-1-Ig). After washing, membranes were probed with HRP-conjugated secondary antibodies for 2 h at room temperature, detected by ECL reagents (C05-07004), and band intensities were quantified.

### 2.10 Cell culture and transfection

The HK2, 786-O, and 769-P cell lines were cultured in DMEM medium (Gibco, C11995500BT) supplemented with 10% fetal bovine serum (HyCyte, FBP-C520) and 1% antibiotics (Servicebio, G4003-100ML) at standard conditions (37°C in a humidified incubator containing 5% CO2 in air). TEK overexpression was achieved using the pcDNA3.1-TEK plasmid, and transfection efficiency was confirmed by RT-qPCR and Western blotting.

### 2.11 Cell proliferation assays

The MTT assay was adopted to measure cell proliferation. Transfected 786-O and 769-P cells were inoculated into appropriate plates, and absorbance was recorded daily for 5 days. For drug treatment groups, cells were seeded into appropriate cultured plate and then treated with 0, 0.5, and 1.0 µM shikonin and 0, 1.25, and 2.5 µM acetylshikonin for 24, 48, and 72h for the time and dose-dependent response assay. Afterward, the related cell proliferation was calculated by the absorbance value at 570 nm. Colony formation assays were also performed, with colonies stained and counted after 14 days to assess long-term proliferation.

### 2.12 Migration and invasion assays

A wound-healing assay was conducted to evaluate cell migration. Scratches were made on the confluent monolayers of transfected cells, and images were captured at 0 and 24 h. Transwell migration and invasion assays were carried out using 24-well chambers coated with or without Matrigel. The migrated or invaded cells were stained, photographed, and quantified.

### 2.13 Differential expression and enrichment analysis

Patients in the E-MTAB-1980 dataset were divided into quartiles based on TEK expression levels. The highest and lowest quartile groups (top and bottom 25 percents) were selected for differential gene expression analysis. Functional enrichment analyses were performed using Gene Set Enrichment Analysis (GSEA), Gene Ontology (GO), and Kyoto Encyclopedia of Genes and Genomes (KEGG) to uncover biological processes and signaling pathways related to TEK expression.

### 2.14 Statistical analysis

Statistical analysis was performed utilizing R software or GraphPad Prism 8. The unpaired t-test was employed to compare the statistical significance of the means, with p < 0.05 considered significant. Significance levels are denoted as follows: *P < 0.05, **P < 0.01, ***P < 0.001.

## 3 Results

### 3.1 MR analysis of ocular diseases and RCC

We assessed the causal association between various ocular diseases and RCC using five algorithms, with the IVW method being predominant.

**FIGURE 2:**
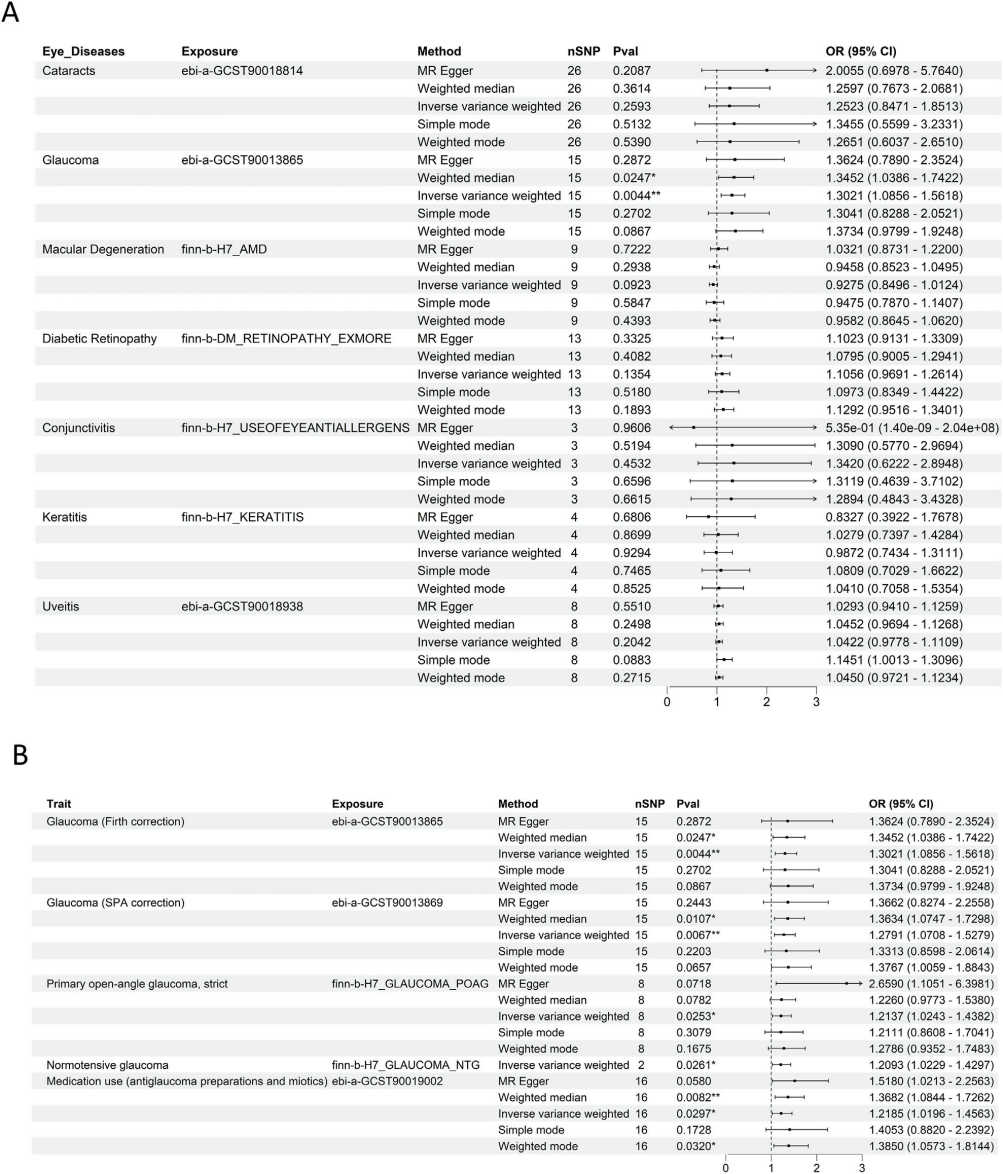
Ocular and glaucoma-RCC association via MR analysis. **(A)** Forest plot depicting the results of five MR methods for evaluating the causal associations between multiple ocular diseases. Glaucoma was significantly associated with an increased risk of RCC compared with other eye diseases. **(B)** Forest plot illustrating the results of five MR methods for assessing the causal relationship between glaucoma and RCC across multiple independent datasets. This validates the robustness of the glaucoma - RCC association by leveraging replication in diverse genetic backgrounds.

The results of IVW and weighted median analyses indicated that glaucoma is significantly associated with an increased risk of RCC, whereas no significant associations were found for other ocular diseases (Figure 2A). Subsequently, the statistically significant association between glaucoma and RCC were further confirmed using multiple datasets [OR = 1.2791, 95%CI (1.0708–1.5279), P = 0.0067] (Figure 2B), supporting a potential causal relationship between them. Detailed results from the five MR methods are provided in Supplementary Table S4.

Cochran’s Q and MR-Egger intercept tests (p > 0.05 for both, Supplementary Table S5) indicated minimal evidence of horizontal pleiotropy and heterogeneity, reinforcing the validity of the MR results. Scatter and funnel plots (Supplementary Figures S1A,B) further supporting the causal association hypothesis. Additionally, the leave-one-out sensitivity analysis demonstrated that the association between glaucoma and RCC remained robust, as it was not influenced by any single SNP (Supplementary Figure S1C).

### 3.2 Analysis based on NHANES database

Given the limited RCC data in the NHANES database and the relation of UC to RCC, UC samples were analyzed to further evaluate the relationship between glaucoma and RCC. The characteristics of each group were detailed in Table 1. Significantly, 15.3% of UC patients developed glaucoma, compared to 6.6% of non-UC patients (p < 0.001). The incidence of UC was significantly higher in males (93.2%) and non-Hispanic whites (60.5%). Hypertension and smoking were more prevalent among UC patients, at 63.8% and 66.1%, respectively. In short, UC was more common among older adults, males, non-Hispanic whites, hypertensive patients, and smokers. The results using weighted data also confirmed the above findings (Supplementary Table S6).

**TABLE 1:** Baseline characteristics of participants with or without urological tumors.

Characteristic	Level	Overall	Non-UC	UC	p
n		7,003	6,826	177	
Gender (%)	Female	3,533 (50.4)	3,521 (51.6)	12 (6.8)	<0.001
	Male	3,470 (49.6)	3,305 (48.4)	165 (93.2)	
Age (mean (SD))		60.54 (12.96)	60.21 (12.90)	73.27 (8.24)	<0.001
Race (%)	Mexican American	1,082 (15.5)	1,070 (15.7)	12 (6.8)	0.002
	Non-Hispanic Black	1,498 (21.4)	1,451 (21.3)	47 (26.6)	
	Non-Hispanic White	3,663 (52.3)	3,556 (52.1)	107 (60.5)	
	Other Hispanic	511 (7.3)	505 (7.4)	6 (3.4)	
	Other Race - Including Multi-Racial	249 (3.6)	244 (3.6)	5 (2.8)	
Education (%)	9-11th grade	1,098 (15.7)	1,073 (15.7)	25 (14.1)	0.657
	College graduate or above	1,355 (19.3)	1,318 (19.3)	37 (20.9)	
	High school graduate	1701 (24.3)	1,665 (24.4)	36 (20.3)	
	Less than 9th grade	1,118 (16.0)	1,086 (15.9)	32 (18.1)	
	Some college or AA degree	1731 (24.7)	1,684 (24.7)	47 (26.6)	
Highbloodpressure (%)	No	3,746 (53.5)	3,682 (53.9)	64 (36.2)	<0.001
	Yes	3,257 (46.5)	3,144 (46.1)	113 (63.8)	
Overweight (%)	No	4,449 (63.5)	4,330 (63.4)	119 (67.2)	0.338
	Yes	2,554 (36.5)	2,496 (36.6)	58 (32.8)	
Smoking (%)	No	3,398 (48.5)	3,338 (48.9)	60 (33.9)	<0.001
	Yes	3,605 (51.5)	3,488 (51.1)	117 (66.1)	
exposure.Glaucoma (%)	No	6,525 (93.2)	6,375 (93.4)	150 (84.7)	<0.001
	Yes	478 (6.8)	451 (6.6)	27 (15.3)	

**FIGURE 3:**
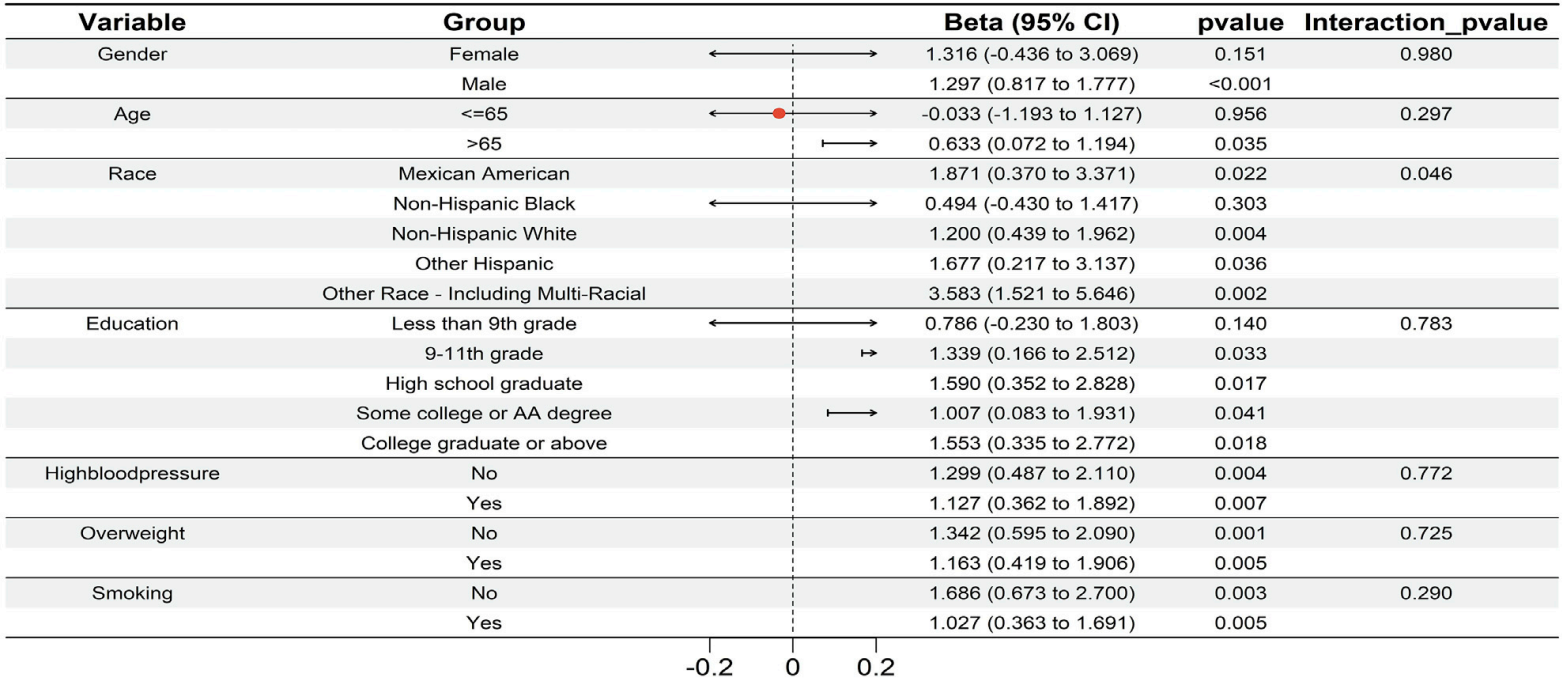
The forest plot of subgroup analysis visualized the robust association between glaucoma and UC across each subgroup (males, aged >65, races except Non - Hispanic Black, education ≥9th–11th grade, and subgroups with/without hypertension, overweight, or smoking; p < 0.05). Race exhibited an interaction effect (p < 0.05), that demographic factors modulate the impact of glaucoma on RCC risk.

Furthermore, the subgroup analysis (Figure 3) showed consistent, significant glaucoma-UC associations in males, aged >65, races except Non-Hispanic Black, education ≥ 9th-11th grade, and subgroups with/without hypertension, overweight, or smoking (p < 0.05). Notably, race exhibited an interaction effect (p < 0.05), suggesting demographic variations in glaucoma’s impact on RCC risk. Together, these findings supported the MR analysis results, reinforcing a potential causal link between glaucoma and RCC and highlighting the need for further research into shared genetic pathways.

### 3.3 Analysis of expression and prognostic correlation of glaucoma-related genes

**FIGURE 4:**
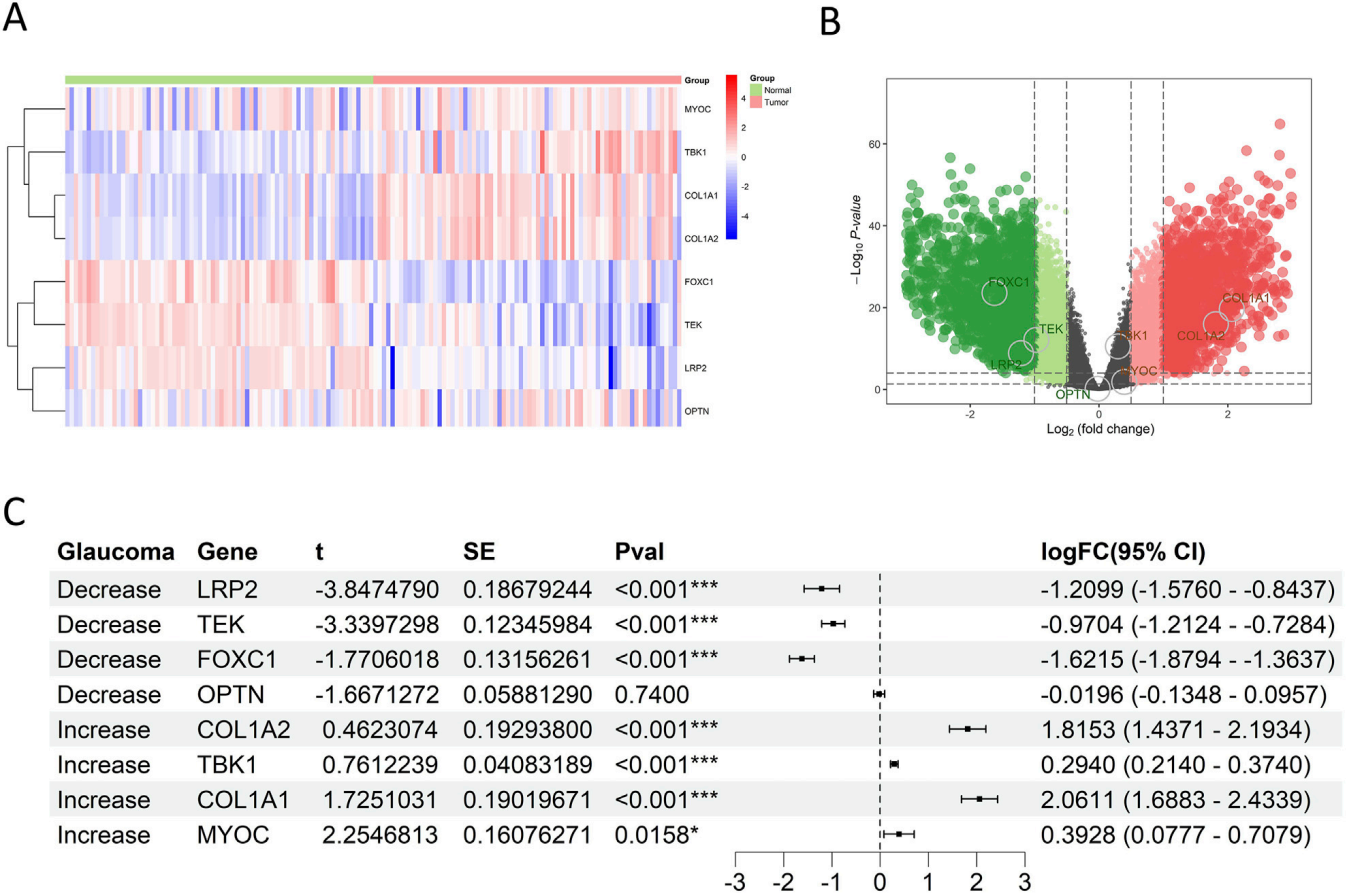
Glaucoma-related gene expression in RCC of GSE53757. **(A)** Heat map showed expression patterns of glaucoma-related genes. Rows = genes, columns = samples; color gradients from red to blue reflected expression levels from high to low, and there was a clear clustering between RCC and normal tissues. **(B)** Volcano plot showed the statistical significance and magnitude of expression changes of glaucoma-related genes in RCC and normal kidney tissues. Genes above the horizontal dashed line had a P value<0.05, indicating significant differential expression. Green dots indicated downregulated genes in RCC, and red dots indicated upregulated genes. **(C)** Forest plot showed that LRP2, TEK and FOXC1 were downregulated, while COL1A2, TBK1, COL1A1 and MYOC were upregulated in RCC tissues.

Through literature review, several key glaucoma-related genes were identified, including MYOC, TBK1, COL1A1, COL1A2, FOXC1, LRP2, OPTN, and TEK. Expression analysis using GSE53757 database revealed significant differences between normal kidney tissues and RCC tissue for all genes except OPTN (Figures 4A–C; Supplementary Figure S2A; Supplementary Table S7). Specifically, MYOC, TBK1, COL1A1, and COL1A2 were upregulated in tumor tissues, while FOXC1, LRP2, and TEK was downregulated (all p < 0.05). These finding are highly in line with the situation in glaucoma.

**FIGURE 5:**
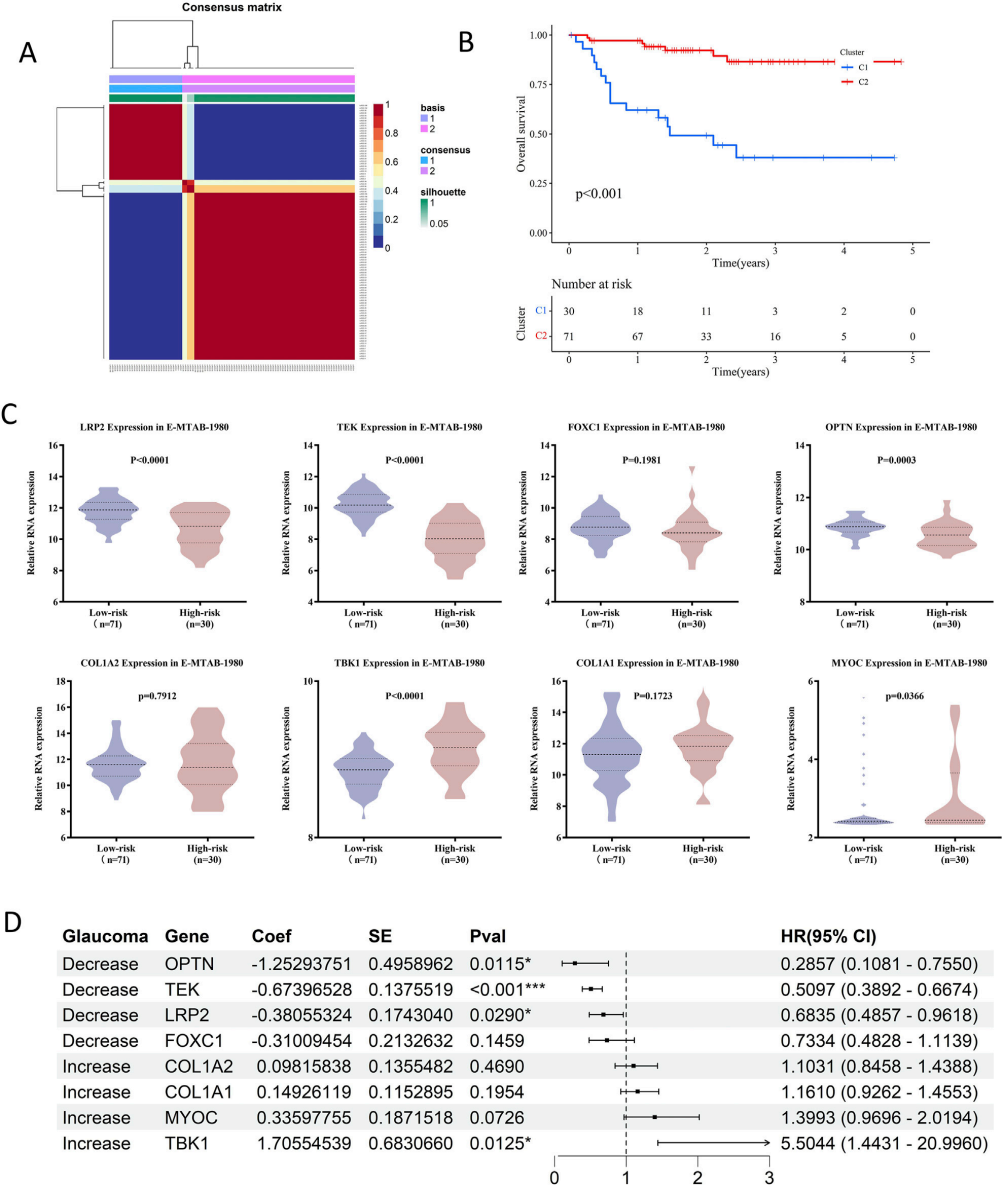
Prognostic impact of glaucoma-related genes on RCC patients in E-MTAB-1980. **(A)** Consensus matrix from NMF clustering, illustrating the stability of assigning RCC patients in E-MTAB-1980. To high-risk and low-risk groups. **(B)** The survival curve analysis showed high-risk patients had significantly shorter overall survival compared to low-risk patients (p < 0.001). The number of patients alive and under observation at each time pointis shown, reflecting the differential impact of the disease on survival in the two groups. **(C)** Violin plots showed that LRP2, TEK and OPTN were significantly downregulated in high-risk groups, while TBK1 and MYOC were upregulated. **(D)** Cox regression analysis in E-MTAB-1980 showed that TEK and LRP2 were protective factors against RCC progression, while MYOC and TBK1 were risk factors.

To further evaluate the association between glaucoma-related genes and prognosis of RCC patients, NMF cluster analysis was first performed to divided RCC patients into high-risk and low-risk groups based on E-MTAB-1980 database (Figures 5A,B; Supplementary Figure S2B). The overall survival (OS) rate of high-risk group was significantly lower than that of low-risk group (P < 0.001). The expression levels of MYOC and TBK1 were upregulated in the high-risk group, while LRP2, OPTN, and TEK were downregulated (all p < 0.05) (Figure 5C; Supplementary Figure S2C; Supplementary Table S8). However, there is no significant difference in the expression levels of COL1A1, COL1A2, and FOXC1 between the two risk groups (all p > 0.05). Moreover, cox regression analysis revealed that high TBK1 expression was significantly associated with poorer prognosis (HR = 5.5044, p = 0.0125), while elevated levels of OPTN, TEK, and LRP2 were correlated to improved survival outcomes (HR = 0.2857, p = 0.0115; HR = 0.5097, p < 0.001; HR = 0.6835, p = 0.0290, respectively) (Figure 5D). Kaplan-Meier survival curves visually represented differences in patient survival based on gene expression levels, illustrating the potential role of glaucoma-related genes in RCC prognosis (Supplementary Figure S2D).

### 3.4 Influence of TEK on the biological behaviors of RCC

**FIGURE 6:**
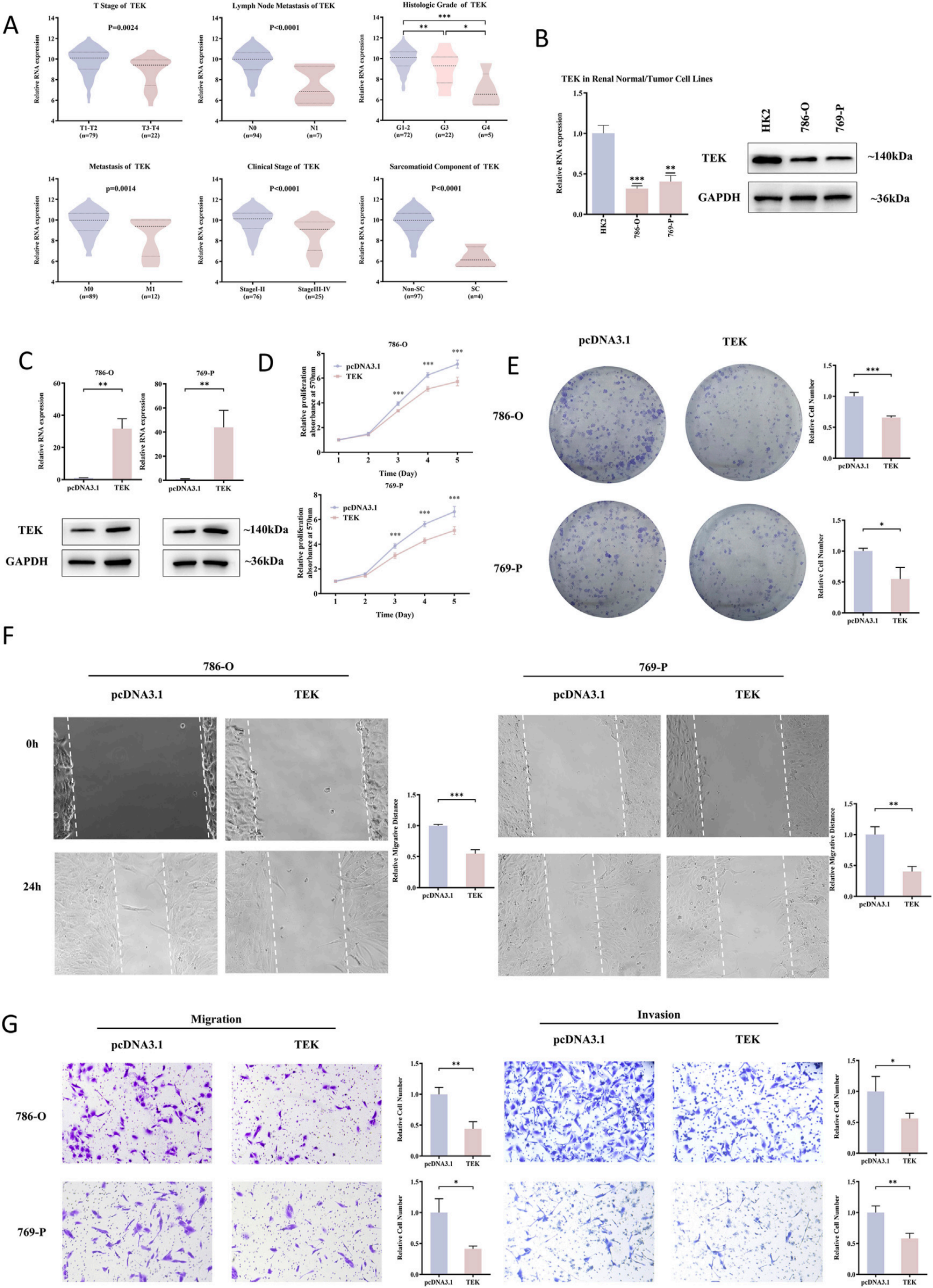
TEK as a tumor suppressor in RCC. **(A)** In the E-MTAB-1980. Dataset, lower TEK expression correlated with advanced tumor stages (T3-T4), lymph node metastasis, higher histologic grades (G3-G4), distant metastasis, late clinical stages (III-IV), and sarcomatoid components. **(B)** TEK mRNA/protein levels were lower in RCC cell lines (786-O, 769-P) than in normal HK2 cells. **(C)** RT-qPCR and Western blot confirmed successful TEK overexpression in 786-O and 769-P cells. **(D)** MTT assays showed that cell proliferation decreased after TEK overexpressed. **(E)** Clone formation assays showed that TEK overexpressed cell proliferation decreased. **(F)** Scratch assays showed that cell metastasis ability decreased after TEK overexpression. **(G)** Transwell assays showed that TEK overexpressed cell metastasis and invasion ability decreased.

Among the genes analyzed, TEK stood out due to its consistent expression patterns and prognostic relevance. E-MTAB-1980 dataset was used to examine the associations between TEK gene expression and clinicopathology of RCC patients. The results (Figure 6A) indicated that lower TEK expression was significantly associated with advanced tumor stage (T3-T4), lymph node metastasis, higher histologic grade (G3-G4), distant metastasis, advanced clinical stage (Stage III-IV), and sarcomatoid component. Moreover, the TEK expression was lower in RCC cell lines (786-O and 769-P) compared to the normal kidney cell line HK2 (Figure 6B). We further performed functional analyses to evaluate the role or TEK, and the overexpression efficiency was successfully confirmed by RT-qPCR and Western blot (Figure 6C). Compared to the control group, the proliferative ability of TEK-overexpressing cells was restricted (Figures 6D,E). Furthermore, TEK-overexpressing cells exhibited an obvious decline in migration and invasion abilities (Figures 6F,G). Together, these findings underscore the potential tumor-suppressive role of TEK in RCC by affecting proliferation, migration, and invasion properties.

### 3.5 Enrichment analysis to predict functions of TEK

GSEA plots demonstrated significant enrichment of the “G2M checkpoint” and “MYC target pathways” in the high TEK expression group (Supplementary Figure S3A), linking TEK to cell cycle control and oncogenic transcriptional regulation, supporting its role as a tumor suppressor in RCC. GO and KEGG analyses revealed that TEK expression was associated with critical structural and regulatory functions within the tumor microenvironment. Specifically, GO analysis highlighted enrichment in “cell adhesion”, “extracellular matrix interaction”, and “vasculature development” (Supplementary Figures S3B–D). KEGG pathways included “ECM-receptor interaction,” “cell cycle,” “complement and coagulation cascades,” and “AGE-RAGE signaling in diabetic complications” (Supplementary Figure S3E), emphasizing the involvement of TEK in immune regulation and ECM dynamics.

### 3.6 Prediction of plant-derived compounds targeting TEK

**FIGURE 7:**
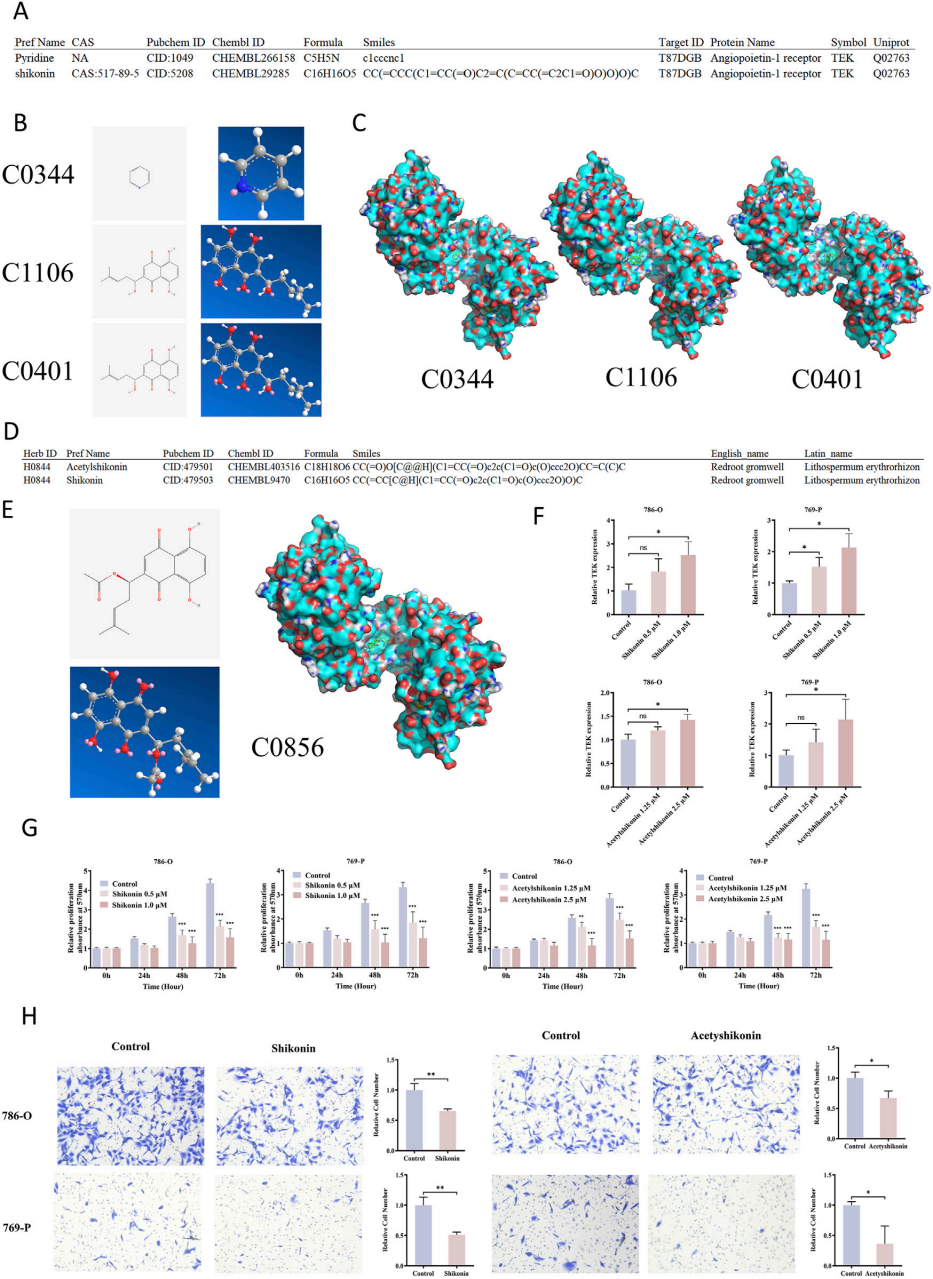
Shikonin and Acetylshikonin inhibit RCC via TEK activation. **(A)** HIT-index database predicted potential TEK-binding plant compounds, including pyridine and shikonin. **(B)** Pyridine (C0344) and shikonin, which has two configurations (C1106 and C0401), were identified, and the 2D/3D structures were retrieved from PubChem. **(C)** Molecular docking showed that pyridine had a low, while shikonin exhibited high binding affinities to TEK. **(D)** Shikonin and acetylshikonin are primary bioactive components of Lithospermum erythrorhizon. **(E)** Molecular docking also revealed that acetyshikonin had a high binding affinity to TEK. **(F)** Treatment of RCC cells with shikonin and acetyshikonin upregulated TEK expression. **(G)** MTT assay demonstrated significant inhibition of cell proliferation after treatment with shikonin and acetyshikonin. **(H)** Transwell migration assay revealed 1.0 μM shikonin and 2.5 μM acetyshikonin reduced cell migration.

Using the HIT-index database, we identified TEK (UniProt ID: Q02763) as a potential target for several plant derived compounds, such as pyridine (ID: C0344, PubChem CID: 1049) and shikonin (Figure 7A). A detailed search on this platform revealed two distinct structural configurations of shikonin (ID: C1106, PubChem CID: 5208; ID: C0401, PubChem CID: 479503). The two-dimensional and three-dimensional structures of these small molecules were obtained from the PubChem database (Figure 7B).

To evaluate their binding potential to TEK, we retrieved the TEK protein structure based on its UniProt ID (Q02763) from the Protein Data Bank (PDB), and performed molecular docking analysis using AutoDock Vina software. The results showed that both configurations of shikonin exhibited strong binding affinities to TEK, with values of −7.5 kcal/mol (C1106) and −7.0 kcal/mol (C0401), respectively, substantially higher than that of pyridine (−3.9 kcal/mol) (Figure 7C; Supplementary Table S9). These high-affinity interactions suggest that shikonin may directly bind to TEK and regulate its function.

Shikonin is primarily derived from Lithospermum, an herb known in traditional medicine (HERB ID: H0844), and its major bioactive components include shikonin and acetylshikonin (ID: C0856, PubChem CID: 479501) (Figure 7D). The two- and three-dimensional structures of acetylshikonin are presented in Figure 7E. Molecular docking further revealed that acetylshikonin also binds strongly to TEK, with a binding affinity of −7.2 kcal/mol (Supplementary Table S9). These findings further suggest that Lithospermum may act as a natural TEK-targeting agent.

### 3.7 Shikonin inhibits RCC cell proliferation and migration by upregulating TEK

We next investigated whether shikonin and acetylshikonin regulate TEK expression in renal cancer cell lines. The qRT-PCR analysis showed that treatment with increasing concentrations of shikonin (0, 0.5, and 1.0 µM) significantly upregulated TEK mRNA levels in both 786-O and 769-P cells in a dose-dependent manner. Specifically, in 786-O cells, 1.0 µM shikonin increased TEK expression by approximately 2.5-fold compared to the control group (P < 0.05), while the lower concentration (0.5 µM) had no significant effect. In 769-P cells, both 0.5 µM and 1.0 µM shikonin significantly increased TEK expression by 1.5- and 2.1-fold, respectively (P < 0.05). Similarly, 2.5 µM acetylshikonin induced a 1.4-fold and 2.1-fold increase in TEK expression in 786-O and 769-P cells, respectively (P < 0.05), whereas the lower concentration (1.25 µM) did not show significant changes (Figure 7F). These findings support a dose-dependent regulation of TEK by shikonin and its derivatives, with more pronounced effects at higher concentrations.

Functionally, MTT assay demonstrated that both shikonin and acetylshikonin significantly inhibited the proliferation of renal cancer cells in a time- and dose-dependent manner. After 48 and 72 h of treatment, cell viability decreased markedly across all tested concentrations (P < 0.001) (Figure 7G). In addition, transwell migration assays revealed that 1.0 µM shikonin reduced the migration of 786-O and 769-P cells by 35% and 50%, respectively (P < 0.01). Similarly, 2.5 µM acetylshikonin suppressed migration by 33% and 64% in the two cell lines (P < 0.01) (Figure 7H). These data collectively indicate that shikonin and acetylshikonin exhibit potent anti-proliferative and anti-migratory effects in RCC cells.

**FIGURE 8:**
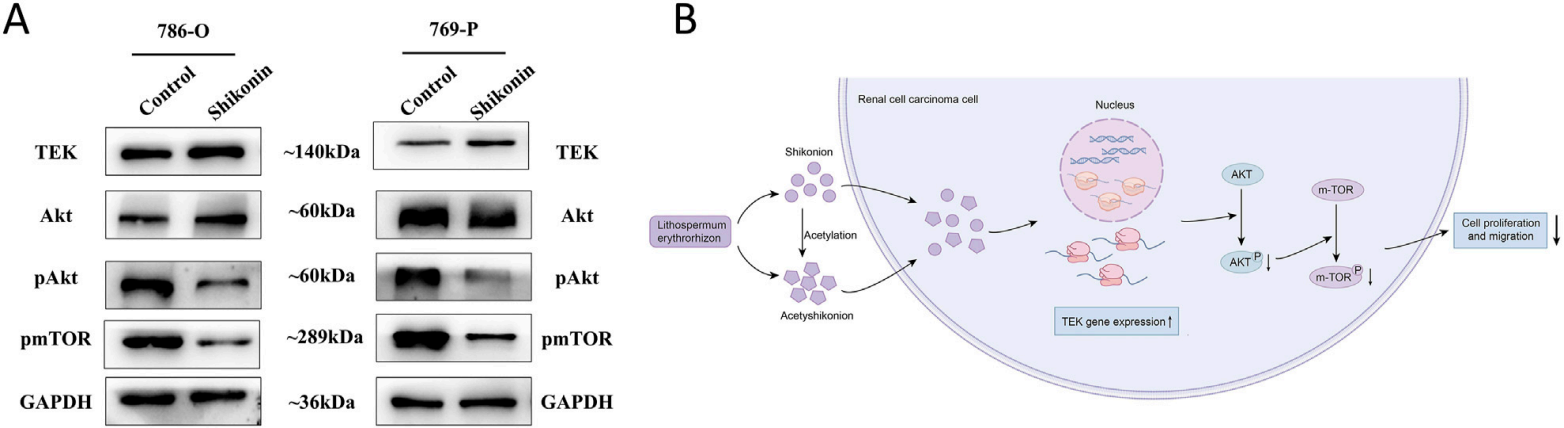
Shikonin regulates TEK and AKT/mTOR pathway in RCC. **(A)** In 786-O and 769-Pcells, shikonin upregulated TEK, with no significant change in total AKT levels, but decreased phosphorylated AKT and mTOR, suggesting shikonin suppresses the AKT/mTOR cascade via TEK. GAPDH ensured equal protein loading. **(B)** Schematic diagram shows that shikonin acts on TEK, which then inhibits AKT/mTOR signaling pathway, which is key to RCC growth.

To explore the underlying molecular mechanisms, Western blot analysis was performed following treatment with 1.0 µM shikonin. Consistent with the qRT-PCR results, TEK protein expression was significantly increased in both 786-O and 769-P cells. Notably, total protein level of AKT remained unchanged, whereas the levels of phosphorylated AKT and phosphorylated mTOR were markedly reduced (Figure 8). Collectively, these findings suggest that shikonin can exerts its anti-tumor effects by binding to and upregulating TEK, which subsequently suppresses the AKT/mTOR signaling pathway (Figure 8).

## 4 Discussion

This study highlights a potential causal relationship between glaucoma and RCC, supported by shared genetic and biological pathways. By employing a two-step MR approach, complemented by NHANES analysis, we present robust evidence linking glaucoma to increased RCC risk. Sensitivity analyses indicated minimal bias due to confounding factors or pleiotropy, reinforcing the reliability of our findings. While glaucoma is often viewed as an ocular disease and RCC as a systemic malignancy, these findings imply that there are common systemic mechanisms at play, opening discussions on shared pathogenic pathways.

Our integrated gene expression analysis strengthens the observed MR association. In the context of glaucoma pathogenesis, research has highlighted several key genes whose altered expression or function is associated with the disease (Selvan et al., 2022; Christ et al., 2015; Kumon et al., 2023; Medina-Trillo et al., 2015). Specifically, elevated expression levels of MYOC, TBK1, COL1A, and COL1A have been observed. Conversely, impaired or diminished functionality of FOXC1, LRP, OPTN, and TEK has also been implicated. Interestingly, these glaucoma-related genes like MYOC, TBK1, COL1A1, COL1A2, FOXC1, LRP2, and TEK also showed significant differential expression in RCC tissues. The upregulation of MYOC and TBK1 in both conditions may indicate involvement in inflammatory and oxidative stress pathways—mechanisms previously implicated in both neurodegeneration and cancer progression (Sears et al., 2019; Ahmad et al., 2016). TBK1 (TANK-binding kinase 1), known for its role in innate immunity and inflammation (Xu et al., 2021), has been linked to oncogenic processes when dysregulated (Wang et al., 2024), potentially contributing to immune evasion and chronic inflammation in RCC. These shared pathways suggest that glaucoma and RCC may share vulnerabilities, potentially targetable with therapies focusing on oxidative stress and inflammation (The Cancer Genome Atlas Research Network, 2013). The overexpression of certain collagen genes (COL1A1 and COL1A2) in RCC suggests a role in extracellular matrix (ECM) remodeling (Devos et al., 2023), a critical factor in tumor metastasis (Piersma et al., 2020), as well as in the structural changes observed in glaucoma (Agarwal and Iezhitsa, 2023). This underscores the potential overlap between ECM-related mechanisms in cancer and ocular diseases. The involvement of ECM remodeling in tumor progression and ocular structural integrity suggests shared molecular disruptions that may be exploited for therapeutic interventions (Sleeboom et al., 2024).

Further survival analysis indicated that high TBK1 expression correlates with poorer RCC prognosis, while increased OPTN, TEK, and LRP2 expression appears protective. TBK1’s high hazard ratio identifies it as a potential therapeutic target (Revach et al., 2020), aligns with recent studies proposing TBK1 inhibitors as potential anti-cancer agents (Miranda et al., 2024; Yang and Liu, 2024). Our findings propose that glaucoma-related genes could serve as potential biomarkers for RCC prognosis and guide the development of targeted therapies.

Among the glaucoma-related genes analyzed, TEK has particularly caught our attention due to its downregulated expression and protective role in patient outcomes. TEK, which encodes the Tie2 receptor tyrosine kinase, is essential for vascular integrity and immune cell regulation (Liao et al., 2021). The activation of TEK in Schlemm’s canal region helps ensure proper fluid drainage (Li et al., 2020), thereby contributing to the prevention of increased intraocular pressure, a key factor in glaucoma pathogenesis. Prior studies have highlighted the importance of TEK in vascular biology and immune regulation, yet its specific role in RCC has not been extensively explored.

Our findings indicate that lower TEK expression correlates with advanced tumor stage, lymph node metastasis, higher histological grade, distant metastasis, and advanced clinical stage in RCC. Functional studies on RCC cell lines confirmed TEK’s role in reduced cell proliferation, migration, and invasion. Enrichment analyses further linked TEK to pathways involved in cell cycle control and transcriptional regulation, including the G2M checkpoint and MYC targets. The G2M checkpoint ensures genomic stability by preventing unregulated cell division (Latif et al., 2001), while MYC drives oncogenic transcriptional programs (Das et al., 2023). Additionally, TEK was found to be associated with immune regulation and extracellular matrix (ECM) remodeling—key processes in the progression of both RCC and glaucoma. Low Tie2 expression, encoded by TEK, has been shown to increase vascular permeability, enhancing inflammatory cell migration (Parikh, 2017) and tumor angiogenesis (Teichert et al., 2017). Conversely, Tie2 activation, regulated by VE-PTP, has a protective effect on the kidneys (Li et al., 2023). Moreover, existing studies have demonstrated that VE-PTP inhibitors can treat glaucoma by activating Tie2 (Brigell et al., 2022). Given these observations, targeting VE-PTP may present a potential therapeutic strategy for RCC.

In recent studies, the therapeutic potential of natural compounds such as shikonin and acetylshikonin has garnered considerable attention. Our experiments have demonstrated that treatment with shikonin and acetylshikonin significantly inhibited the proliferation and migration of RCC cells, concurrently inducing a marked upregulation of TEK mRNA and protein expression. This finding is consistent with the tumor-suppressive role of TEK in RCC, where low TEK expression is associated with advanced tumor staging, lymph node metastasis, and poor prognosis. Notably, while shikonin treatment upregulates TEK, it also significantly inhibits the phosphorylation levels of AKT and mTOR, suggesting that TEK may act as a negative regulator of AKT activity. It is worth mentioning that previous studies have found that TEK knockdown can significantly promote AKT phosphorylation and inhibit the apoptosis of renal cancer cells by upregulating the downstream pro-apoptotic proteins Bcl-2 and Bcl-xL (Chen et al., 2021). Consistent with this regulatory relationship, a study in colorectal cancer indicated that macrophage-derived SHP-2 can inhibit TEK protein phosphorylation, thereby inactivating the PI3K/AKT/mTOR pathway and suppressing metastasis (Wu et al., 2023). Furthermore, our molecular docking results indicated high-affinity binding of both shikonin and acetylshikonin to the TEK protein. Collectively, these results suggest that shikonin exerts its anticancer effects, at least in part, by inhibiting the AKT/mTOR signaling pathway via TEK.

Shikonin has been shown to modulate key AKT/mTOR downstream effectors, including p70S6K/4E-BP1/eIF4E, suppressing HIF-1α synthesis and leading to cell cycle arrest alongside reduced expression of cell cycle-related proteins (Li et al., 2017). Our GSEA analysis revealed that the high TEK expression group was enriched in pathways related to the “G2M checkpoint.” This is reinforced by established evidence that pharmacological inhibition of AKT/mTOR phosphorylation can induce G2/M phase arrest and apoptosis in cancer cells (Li et al., 2024), further supporting the notion that shikonin can regulate the balance between apoptosis and survival in renal cancer cells through the TEK-AKT/mTOR axis. Multiple studies corroborate shikonin’s efficacy in inhibiting the AKT/mTOR pathway (Chen et al., 2014; Guo et al., 2019; Han et al., 2025), explaining its potent antitumor activity and also offering novel strategies to overcome targeted therapy resistance. For instance, in AKT inhibitor-resistant cells, shikonin may enhance or restore pathway inhibition by upregulating TEK expression. Shikonin demonstrates synergistic effects when combined with the PI3K-Akt-mTOR inhibitor BEZ235, significantly reducing the viability of chemotherapy-resistant lung cancer cells (Huang et al., 2024). Furthermore, in sunitinib-resistant RCC cells, shikonin induces necroptosis by suppressing AKT/mTOR signaling and activating the necrosome complex (Mar et al., 2022). For ccRCC patients undergoing ipilimumab treatment, combination therapy with shikonin has been identified as a promising novel strategy (Lyu et al., 2023).

As the main active component of Lithospermum erythrorhizon, shikonin has been shown to possess anti-inflammatory, antioxidant, and anti-angiogenic properties (Sun et al., 2022). Notably, the downregulation of TEK expression also plays a key role in the pathology of glaucoma. Therefore, shikonin, by upregulating TEK expression, may act on the shared pathological mechanisms of glaucoma and RCC. Moreover, shikonin has been shown to alleviate oxidative stress and neurodegenerative damage associated with glaucoma (Kang et al., 2021), which may be achieved through the inhibition of the AKT/mTOR pathway. This hypothesis warrants further validation using animal models.

Despite these significant findings, several limitations should be acknowledged. First, the MR analysis mainly utilized data from European populations, which may limit its applicability to other ethnic groups. Second, the NHANES dataset lacked detailed RCC-specific information, so we used urological cancer as a proxy outcome, which may not fully reflect RCC biology. Third, while our findings suggest that TEK mediates the anti-tumor effects of shikonin, the exact molecular mechanism, such as how shikonin upregulates TEK or enhances its interaction with downstream effectors, remains unclear and requires further investigation. In addition, although shikonin and acetylshikonin show strong anticancer activity *in vitro*, their effectiveness and safety *in vivo* need to be evaluated in preclinical models before clinical translation is possible. In conclusion, our study provides novel insights into the link between glaucoma and RCC and highlights TEK as a potential therapeutic target. However, further mechanistic and translational studies are needed to fully validate these findings.

## Data Availability

The data generated in this study are available within the article and its supplementary data files. GWAS data in this study can be got from the NHGRI-EBI GWAS Catalog (https://www.ebi.ac.uk/gwas/), and FinnGen (https://www.finngen.fi/en). Expression profile data analyzed were obtained from Gene Expression Omnibus (GEO) at GSE53757 (https://www.ncbi.nlm.nih.gov/geo/query/acc.cgi?acc=GSE53757) and E-MTAB-1980 (https://www.ebi.ac.uk/arrayexpress/experiments/E-MTAB-1980/).
