# Supplementary material for: Shikonin as a therapeutic agent in renal cell carcinoma: insights from TEK-related causal association with glaucoma
Source: Front Pharmacol. 2025 Jul 30;16:1580704. doi: 10.3389/fphar.2025.1580704 (PMC12343566; doi:10.3389/fphar.2025.1580704)
Supplement: Supplementary file 1 [file DataSheet2.pdf]

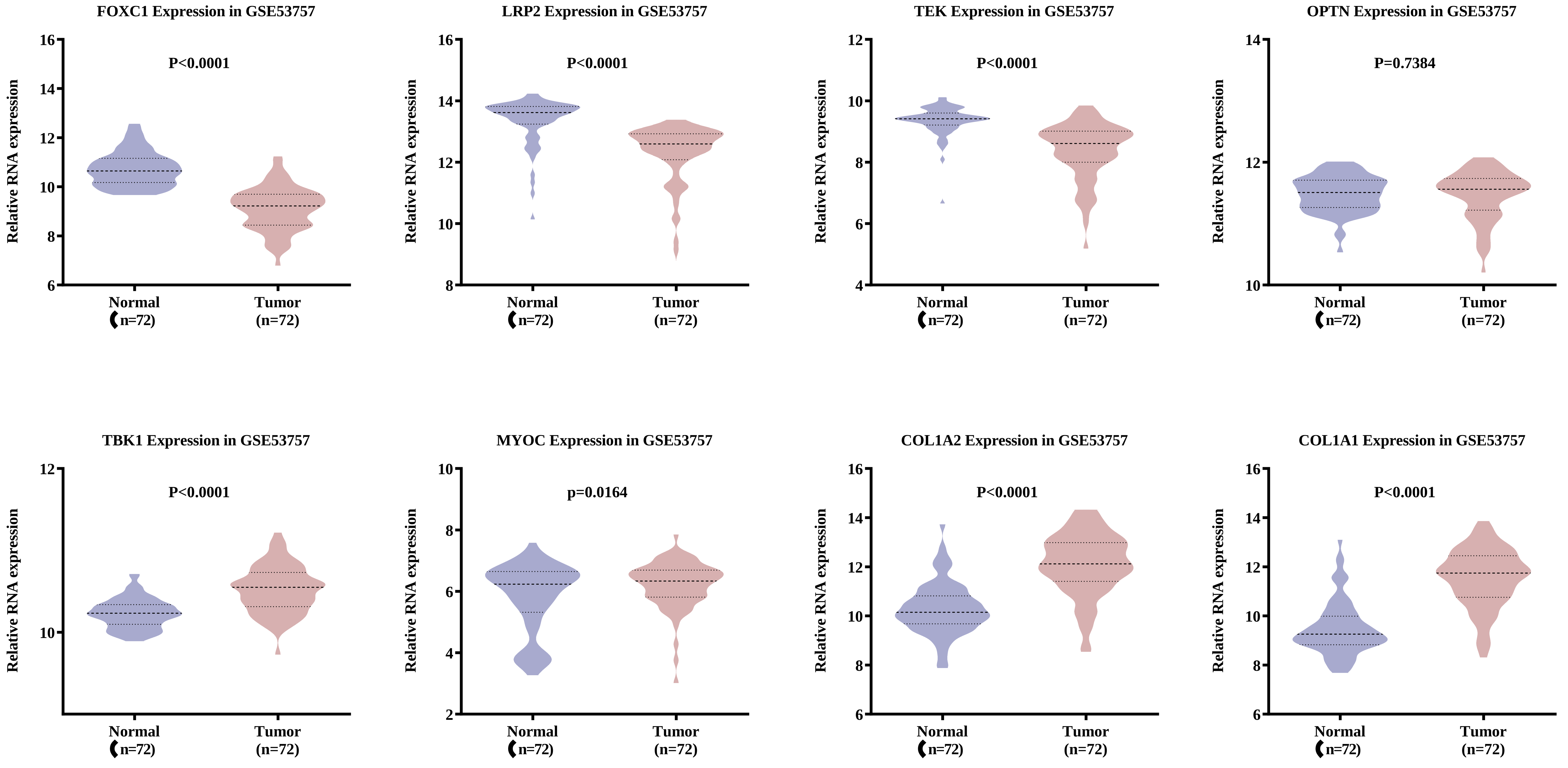

Figure S2. (**A**) Expression of glaucoma-related genes in GSE53757 tumors and normal tissues.

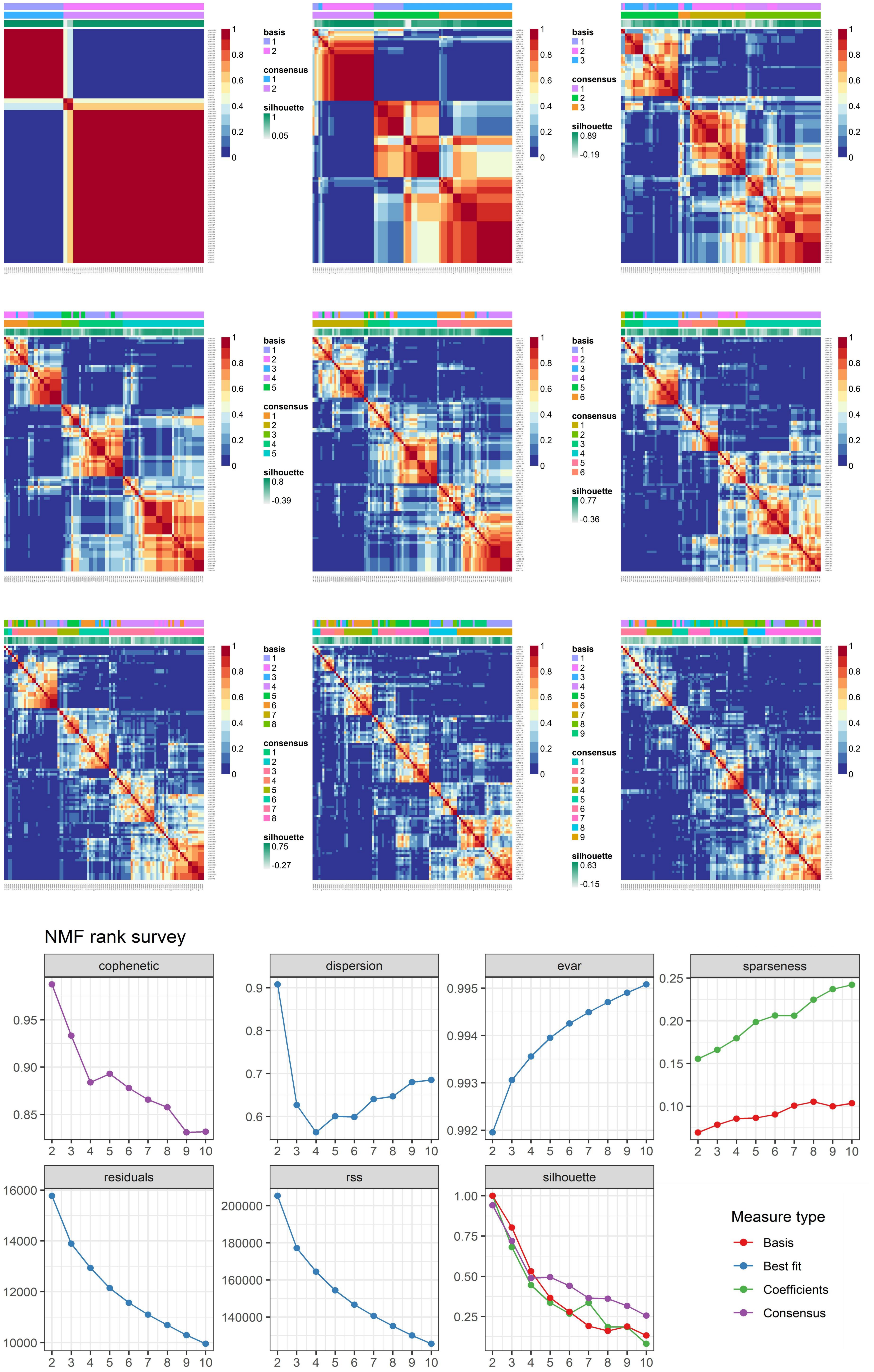

Figure S2. (**B**) Diagram of the process of grouping E-MTAB-1980 using NMF cluster analysis.

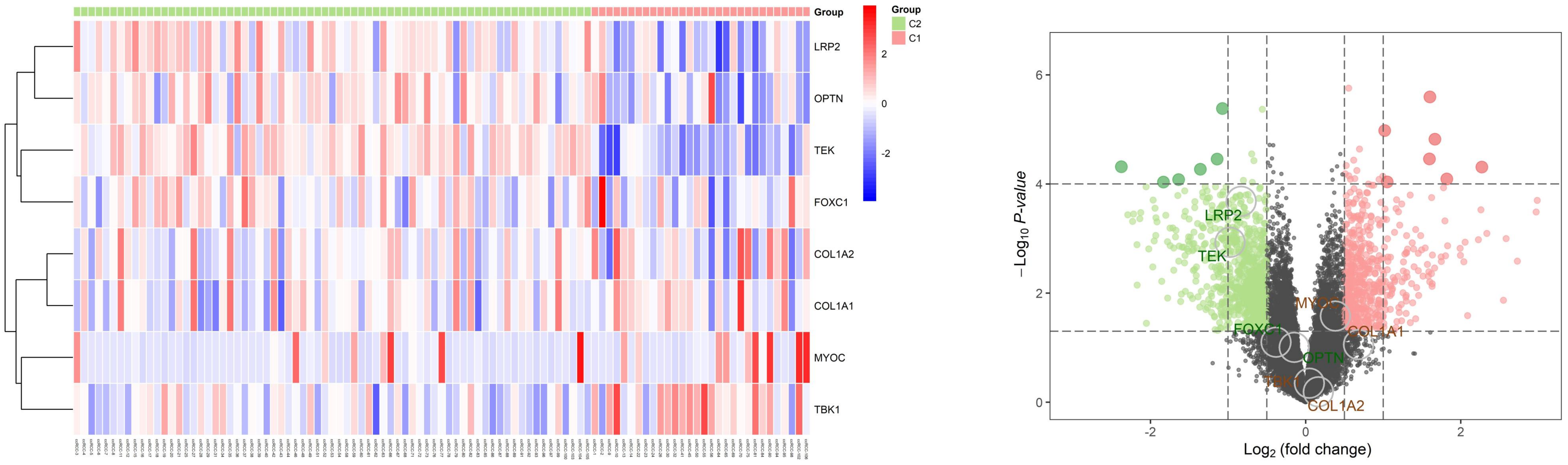

Figure S2. (**C**) Heat map and volcano map of glaucoma-related gene expression in the E-MTAB-1980 after grouping using NMF.

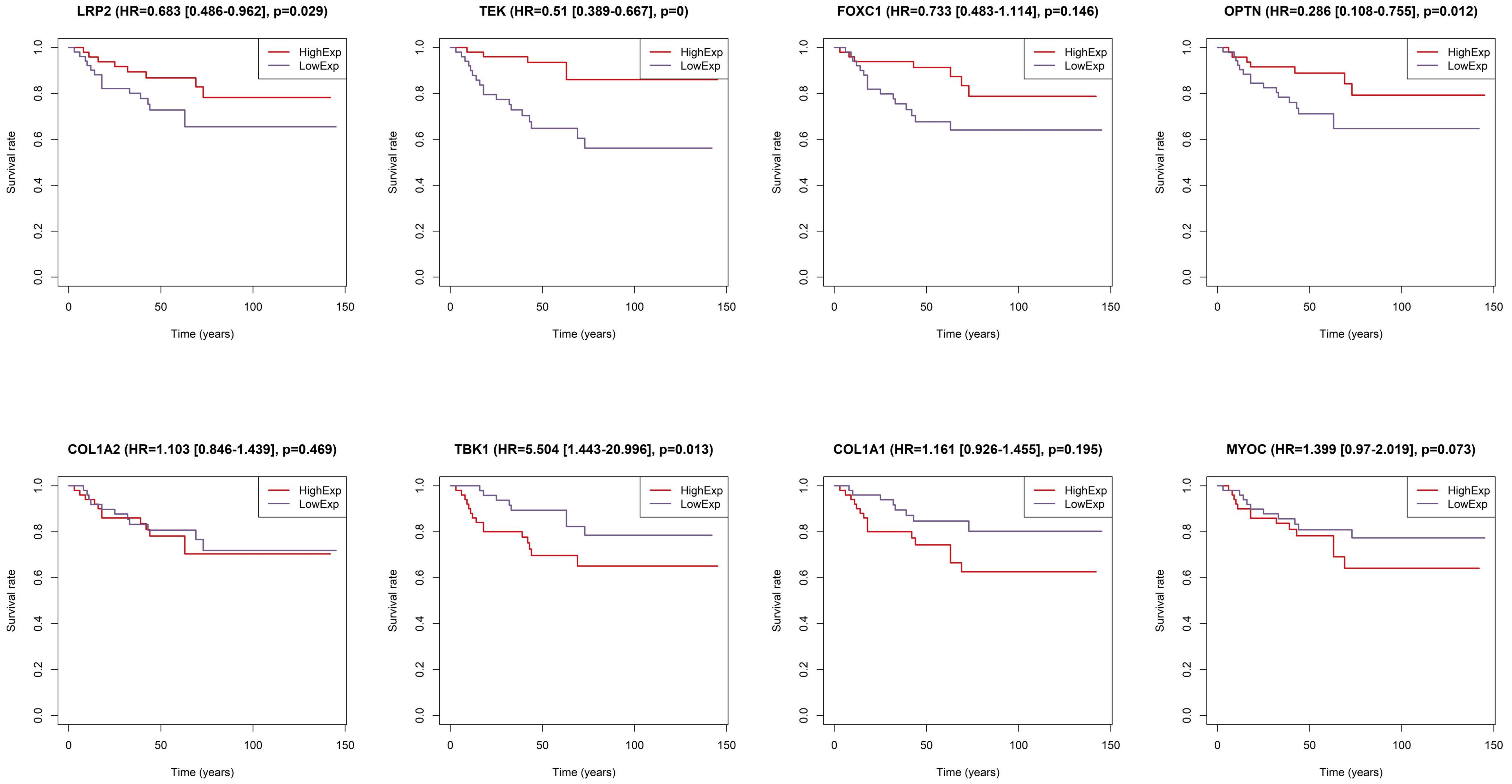

Figure S2. (**D**) The Kaplan-Meier survival curve of glaucoma-related genes expressed in E-MTAB-1980 .
