# Supplementary material for: Shikonin as a therapeutic agent in renal cell carcinoma: insights from TEK-related causal association with glaucoma
Source: Front Pharmacol. 2025 Jul 30;16:1580704. doi: 10.3389/fphar.2025.1580704 (PMC12343566; doi:10.3389/fphar.2025.1580704)
Supplement: Supplementary file 2 [file DataSheet3.pdf]

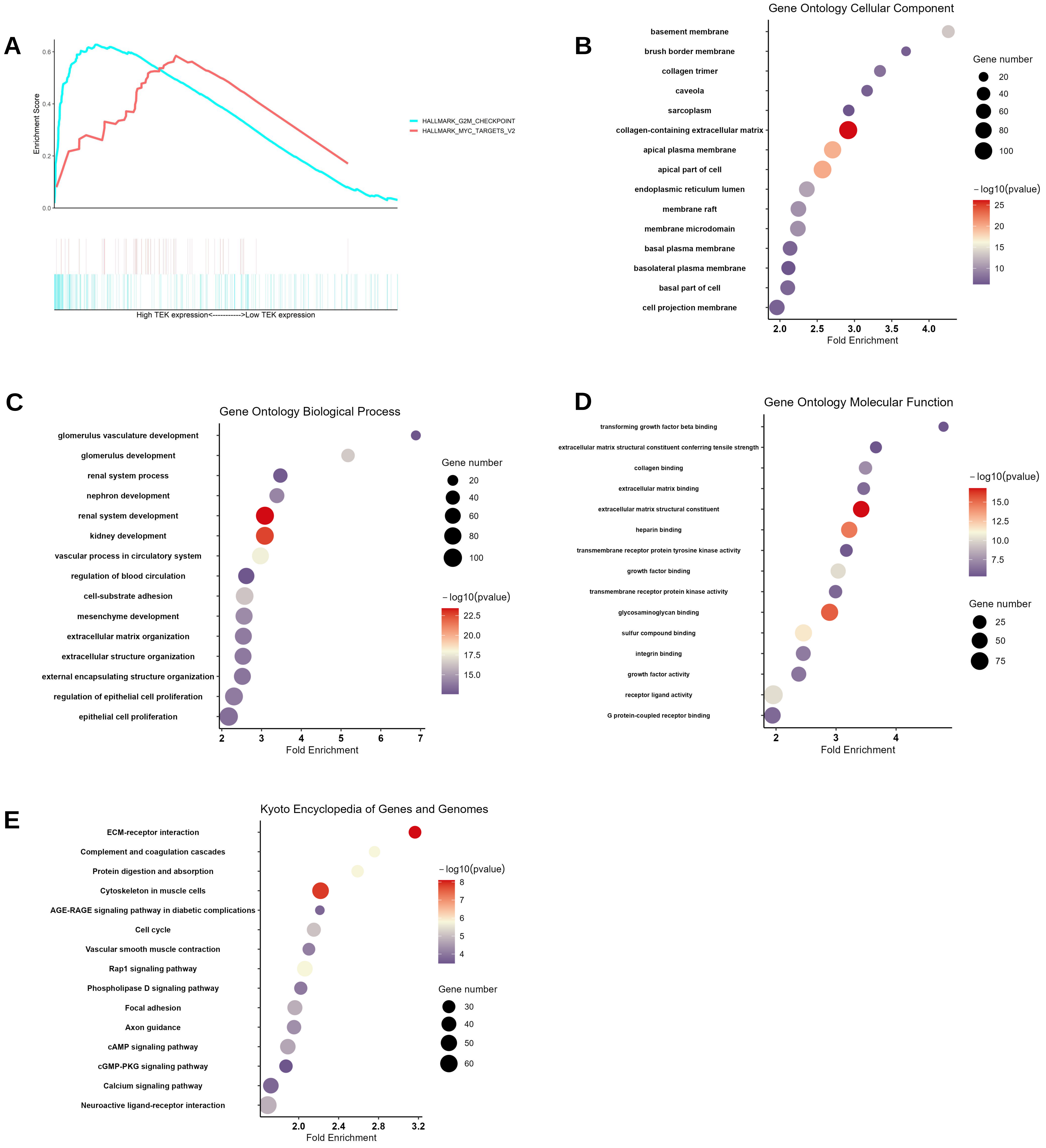

Figure S3. Enrichment analysis plots of differential genes between high-expression and low-expression groups of TEK. Enrichment plots of GSEA analysis (**A**), GO analysis (**B-D**) and bubble plots of KEGG pathway enrichment analysis (**E**)for the differential genes between the two groups of TEK expression.
