# Supplementary material for: Shikonin as a therapeutic agent in renal cell carcinoma: insights from TEK-related causal association with glaucoma
Source: Front Pharmacol. 2025 Jul 30;16:1580704. doi: 10.3389/fphar.2025.1580704 (PMC12343566; doi:10.3389/fphar.2025.1580704)
Supplement: Supplementary file 3 [file DataSheet1.pdf]

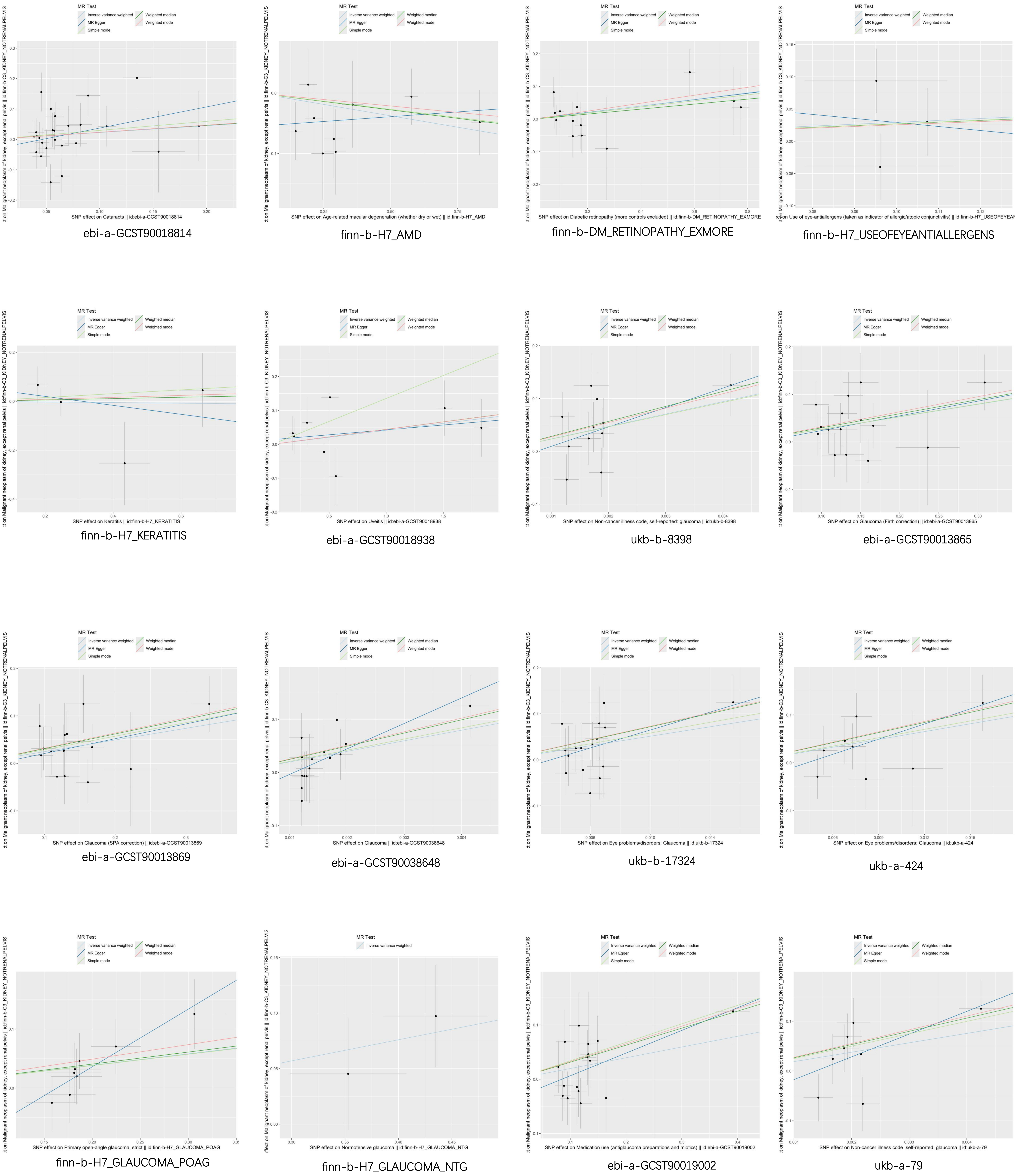

Figure S1. (**A**)The scatter plots of the results of two-step MR analysis.

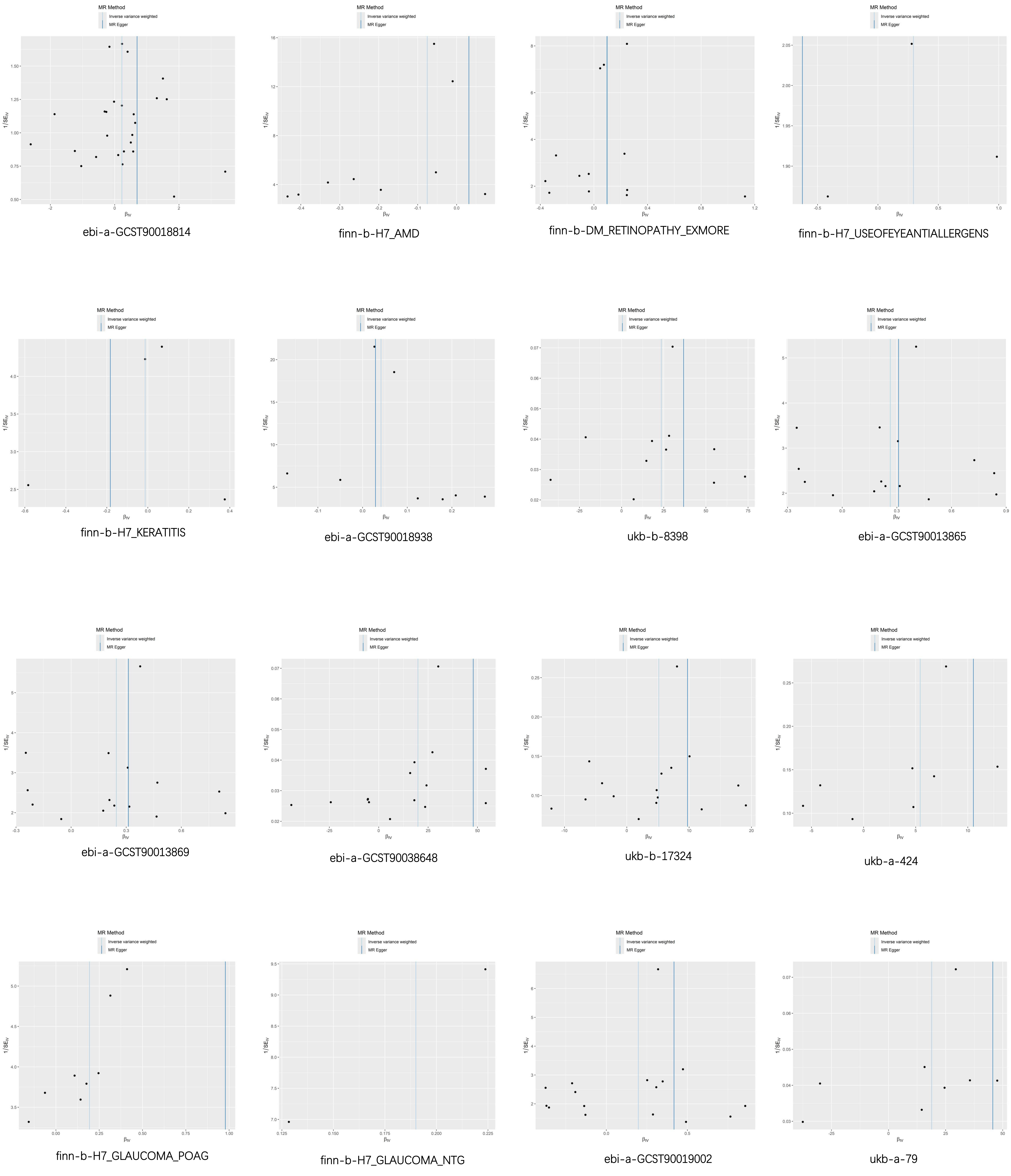

Figure S1. (**B**) The funnel plots of the results of two-step MR analysis.

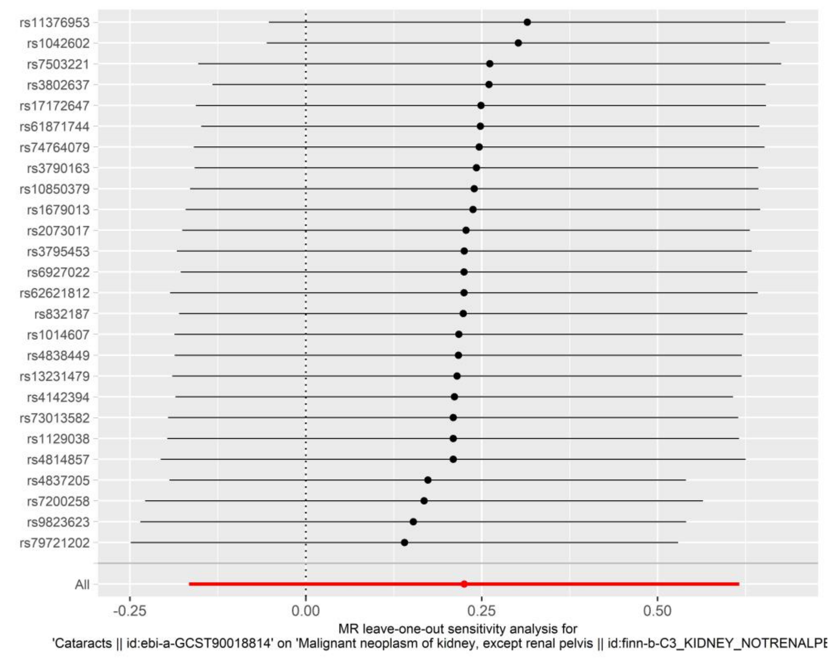

ebi-a-GCST90018814

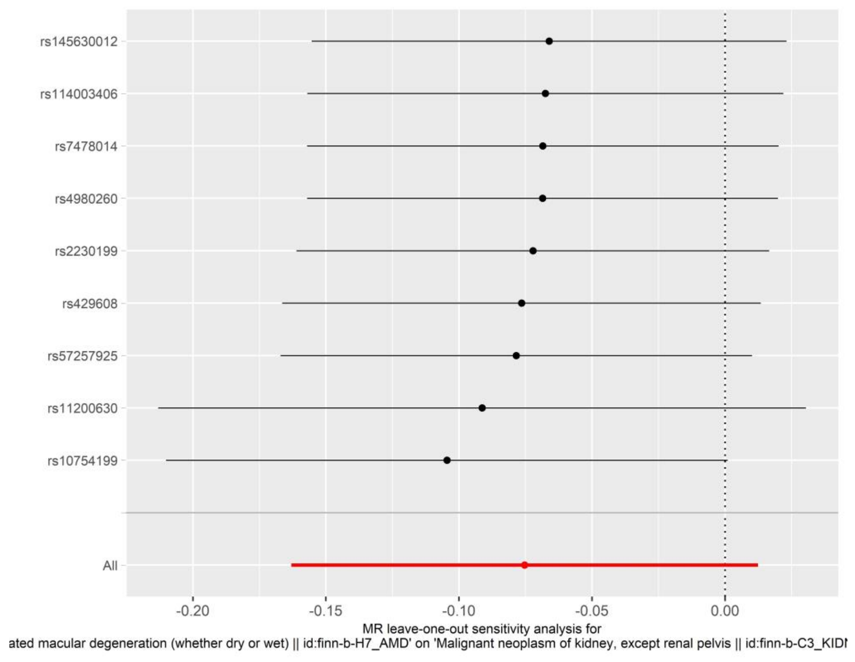

finn-b-H7_AMD

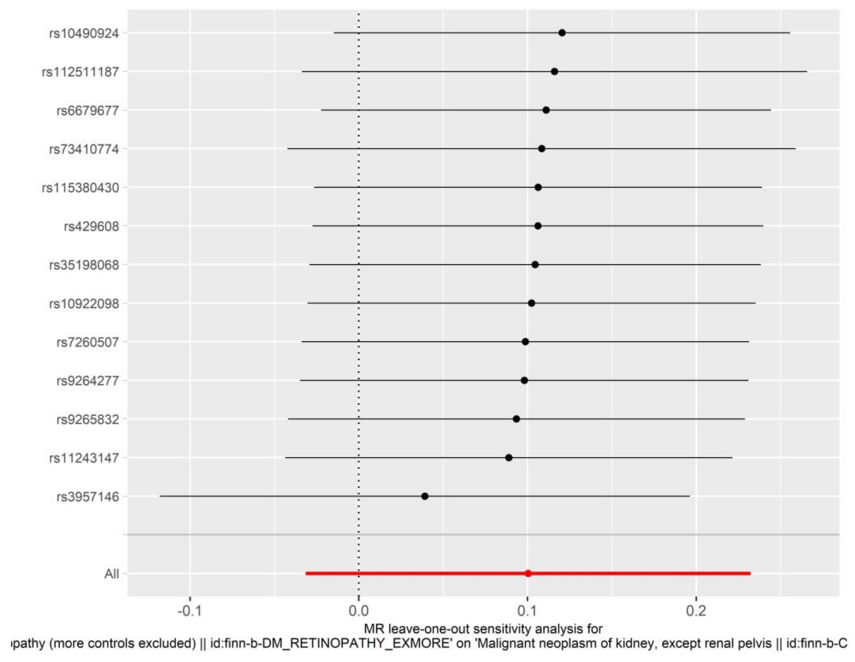

finn-b-DM_RETINOPATHY_EXMORE

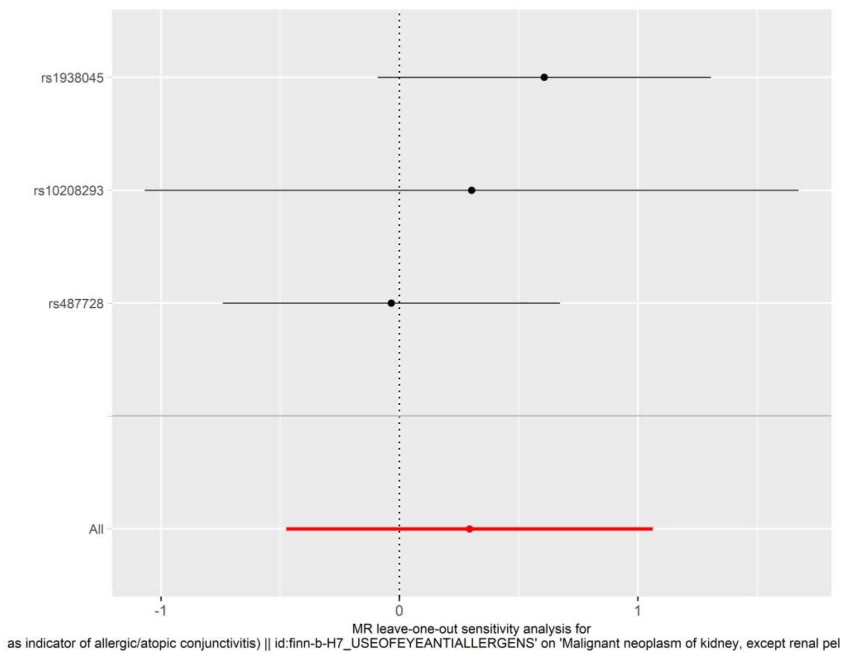

finn-b-H7_USEOFEYEANTIALLERGENS

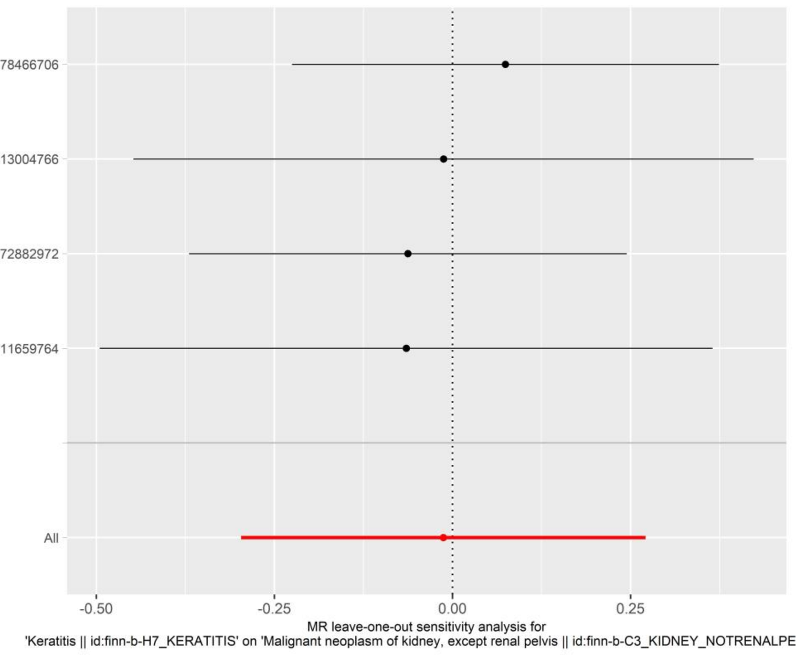

finn-b-H7_KERATITIS

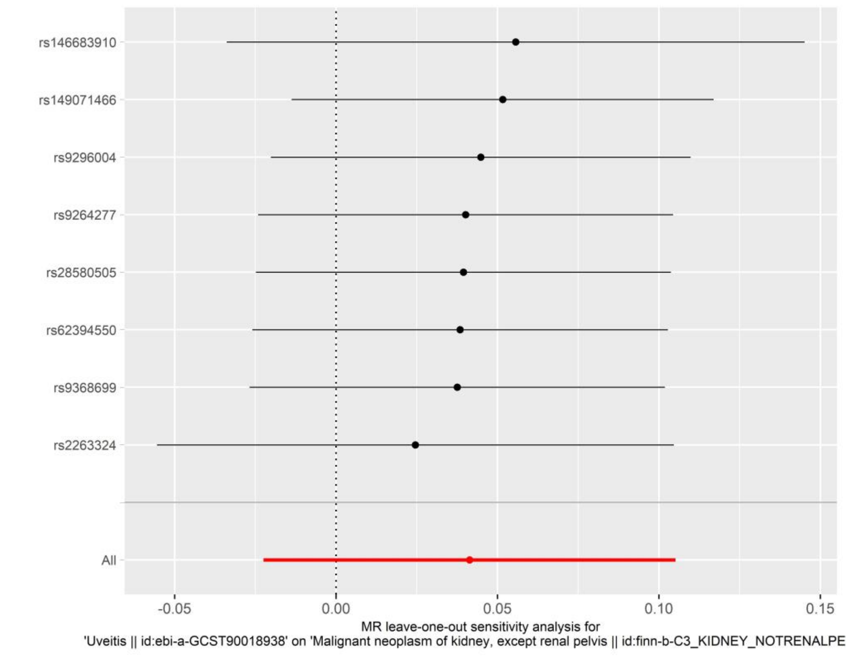

ebi-a-GCST90018938

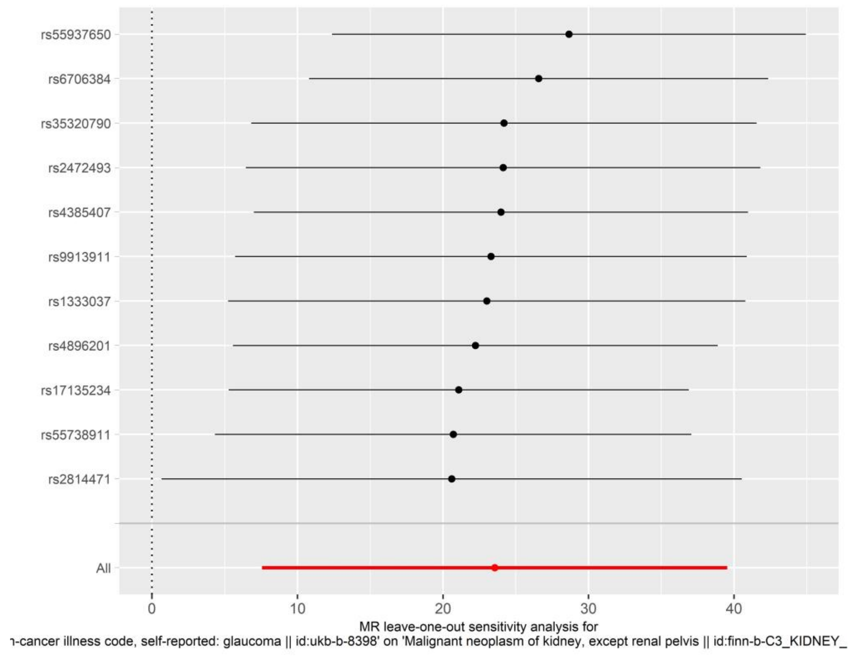

ukb-b-8398

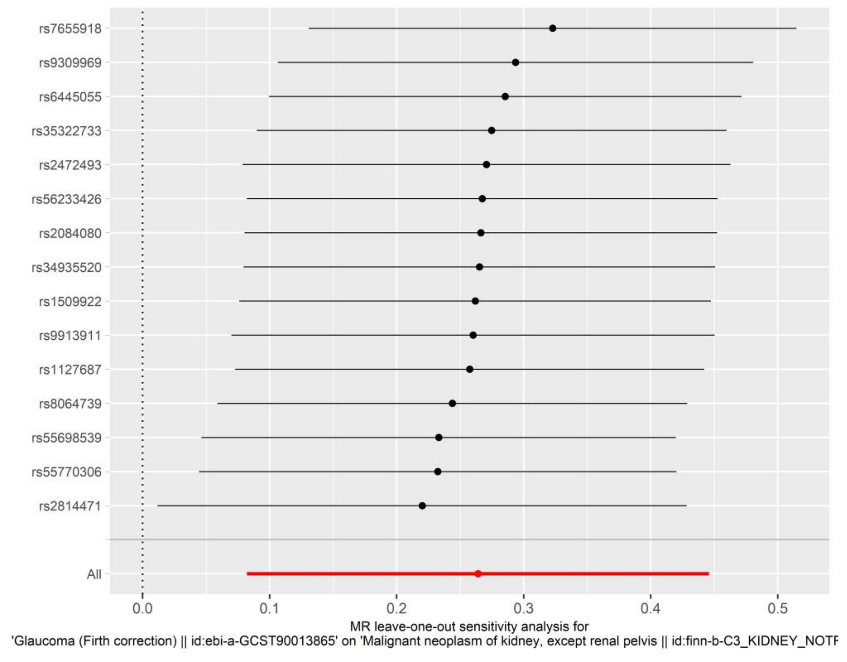

ebi-a-GCST90013865

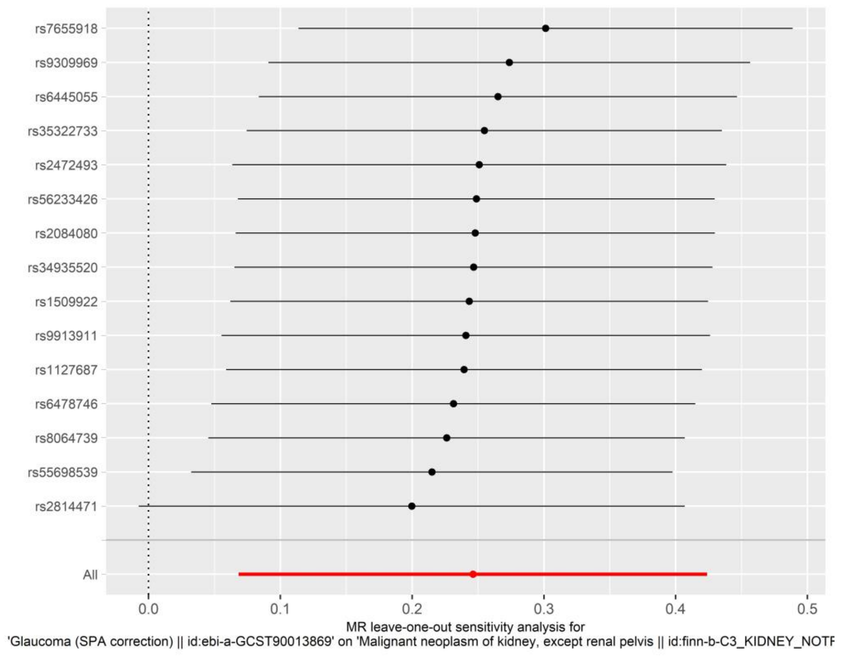

ebi-a-GCST90013869

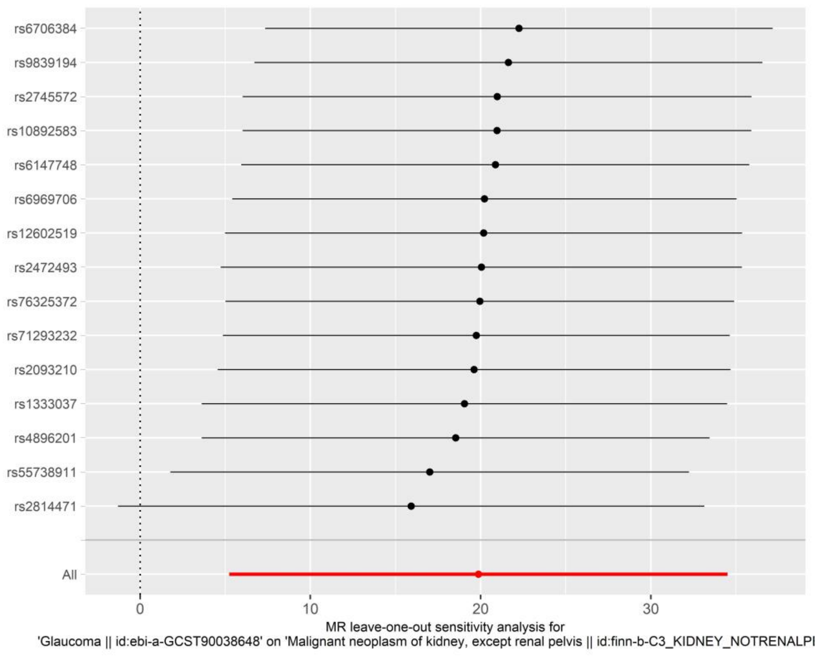

ebi-a-GCST90038648

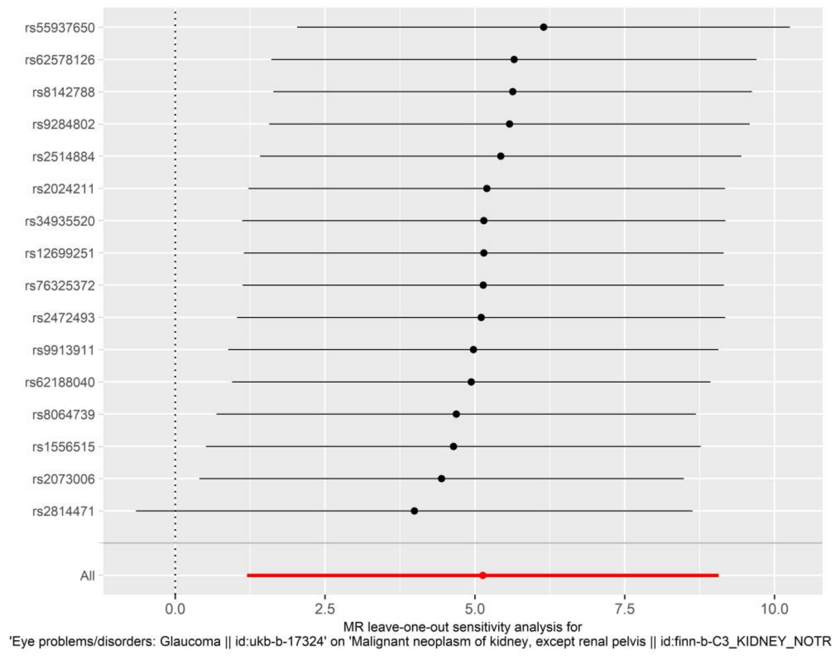

ukb-b-17324

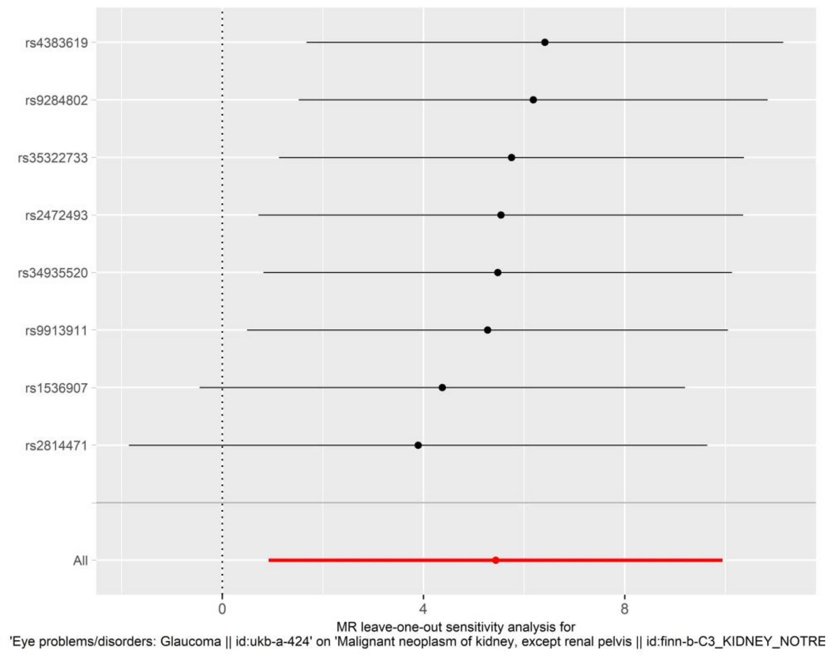

ukb-a-424

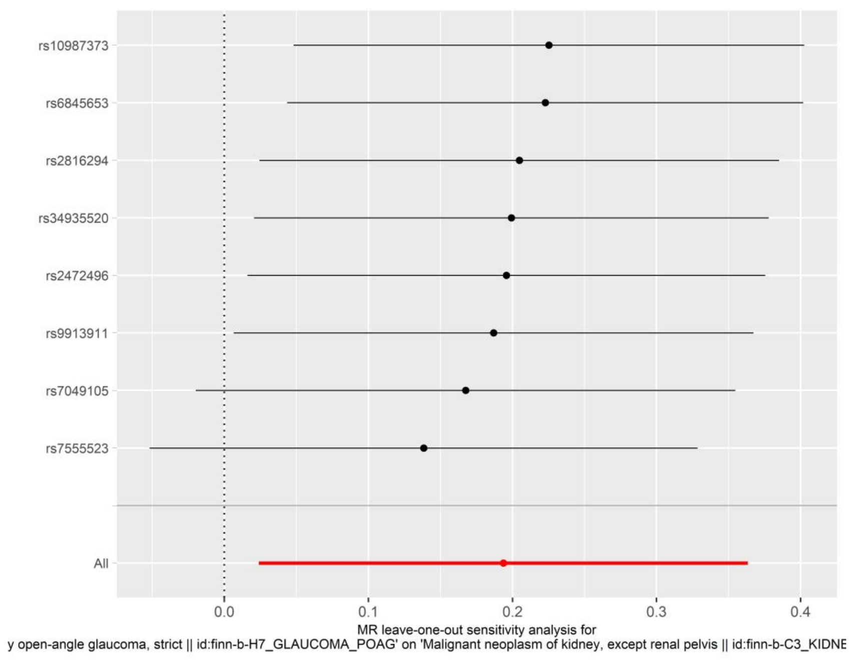

finn-b-H7_GLAUCOMA_POAG

finn-b-H7_GLAUCOMA_NTG

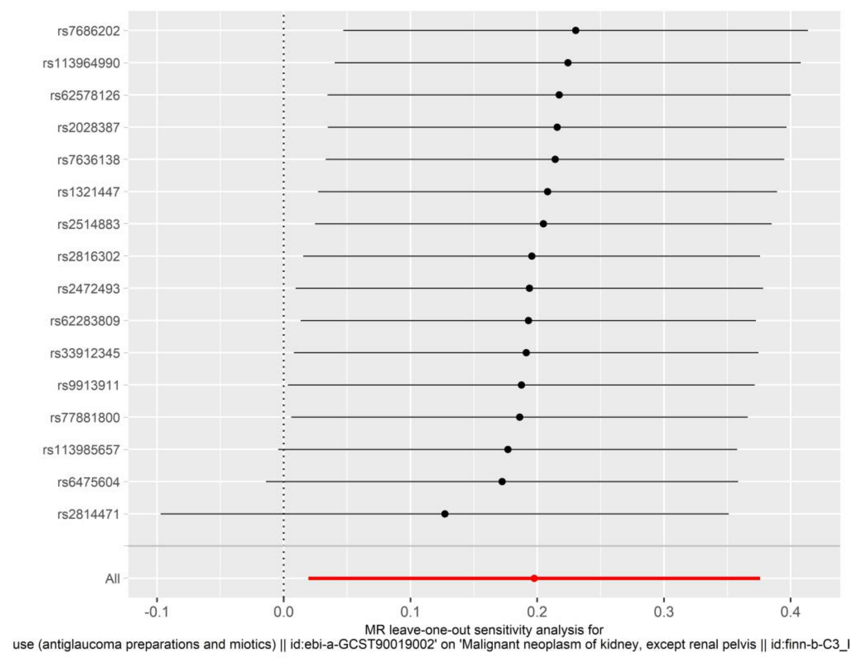

ebi-a-GCST90019002

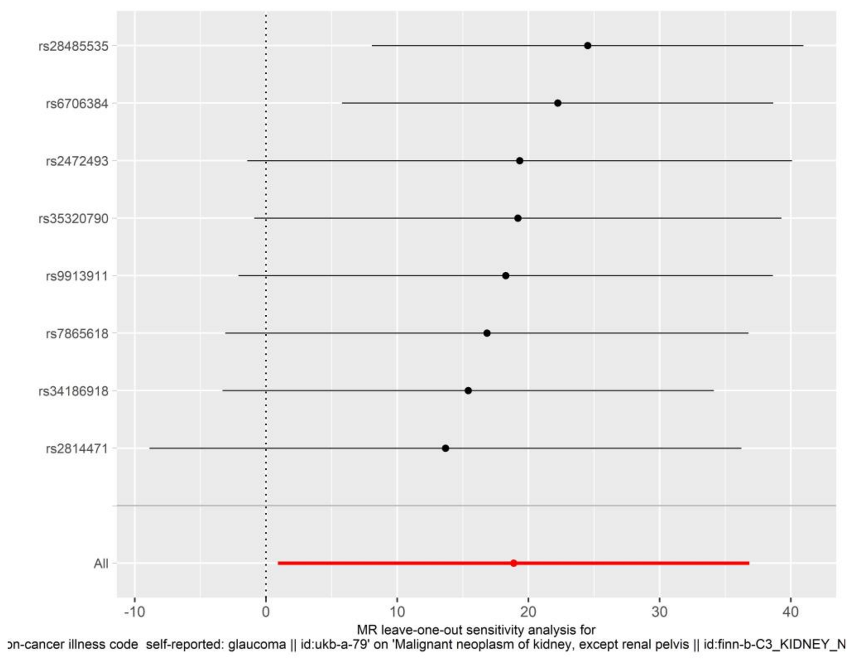

ukb-a-79

Figure S1. (**C**) The results of leave-one-out sensitivity analysis in two-step MR analysis.
